# Studies into the mechanism of measles-associated immune suppression during a measles outbreak in the Netherlands

**DOI:** 10.1038/s41467-018-07515-0

**Published:** 2018-11-23

**Authors:** Brigitta M. Laksono, Rory D. de Vries, R. Joyce Verburgh, Eline G. Visser, Alwin de Jong, Pieter L. A. Fraaij, Wilhemina L. M. Ruijs, David F. Nieuwenhuijse, Henk-Jan van den Ham, Marion P. G. Koopmans, Menno C. van Zelm, Albert D. M. E. Osterhaus, Rik L. de Swart

**Affiliations:** 1 Department of Viroscience, Postgraduate School of Molecular Medicine, Erasmus MC, University Medical Centre Rotterdam, Wytemaweg 80, 3015 CN Rotterdam, The Netherlands; 2 Department of Paediatrics, Postgraduate School of Molecular Medicine, Erasmus MC, University Medical Centre Rotterdam, Wytemaweg 80, 3015 CN Rotterdam, The Netherlands; 3 Centre for Infectious Disease Control, National Institute for Public Health and the Environment (RIVM), Antonie van Leeuwenhoeklaan 9, 3721 MA Bilthoven, The Netherlands; 4 Department of Immunology, Postgraduate School of Molecular Medicine, Erasmus MC, University Medical Centre Rotterdam, Wytemaweg 80, 3015 CN Rotterdam, The Netherlands; 5 Present Address: ENPICOM BV, ′s-Hertogenbosch, 5211 AX The Netherlands; 6 Present Address: Department of Immunology and Pathology, Monash University, and The Alfred Hospital, Melbourne, VIC 3004 Australia; 7 Present Address: Research Centre for Emerging Infections and Zoonoses, University of Veterinary Medicine (TiHo-RIZ), Hannover, 30559 Germany

## Abstract

Measles causes a transient immune suppression, leading to increased susceptibility to opportunistic infections. In experimentally infected non-human primates (NHPs) measles virus (MV) infects and depletes pre-existing memory lymphocytes, causing immune amnesia. A measles outbreak in the Dutch Orthodox Protestant community provided a unique opportunity to study the pathogenesis of measles immune suppression in unvaccinated children. In peripheral blood mononuclear cells (PBMC) of prodromal measles patients, we detected MV-infected memory CD4^+^ and CD8^+^ T cells and naive and memory B cells at similar levels as those observed in NHPs. In paired PBMC collected before and after measles we found reduced frequencies of circulating memory B cells and increased frequencies of regulatory T cells and transitional B cells after measles. These data support our immune amnesia hypothesis and offer an explanation for the previously observed long-term effects of measles on host resistance. This study emphasises the importance of maintaining high measles vaccination coverage.

## Abstract (web summary)

The mechanisms by which measles virus infection induces transient immune suppression in humans are poorly understood. Here, Laksono and colleagues characterise the pathogenesis of measles-associated immune suppression in unvaccinated children, and shed new light on the long-term effects of measles on the host.

## Introduction

Measles virus (MV) is a highly infectious virus that is transmitted through aerosols and droplets and causes measles. Measles is characterised by fever, cough and a maculopapular skin rash^1^. The disease is associated with a transient immune suppression and increased risk of childhood morbidity and mortality for a period of more than 2 years^2,3,4^. Paradoxically, measles also induces strong cellular and humoral immune responses that mediate lifelong protection^5^. Despite the availability of safe and effective live-attenuated vaccines, measles and its sequelae still cause more than 85,000 deaths globally^6^. In Europe, a four-fold increase in the number of measles cases was reported in 2017, largely due to declining vaccination coverage^7^. The increase of vaccine hesitancy is a major concern, since it appears to be linked to the lack of understanding of the impact of measles as serious childhood disease. To reach the goals set out in the Global Vaccine Action Plan, which include elimination of measles by 2020 in five out of six regions of the World Health Organisation (WHO)^8^, improvement of public health information, education and communication is crucial. A better understanding of the pathogenesis of measles and measles-associated immune suppression will help convey the message that vaccination is vital.

MV infects cells after binding to cellular receptors CD150 or nectin-4, expressed on subsets of immune cells or the adherens junctions of epithelial cells, respectively^9–11^. In experimentally infected non-human primates (NHPs), MV initially targets myeloid cells in the respiratory tract, which act as Trojan horses by transmitting MV to CD150^+^ lymphocytes in lymphoid tissues, leading to viraemia and systemic virus dissemination^12–15^. Ensuing lymphocyte depletion and follicular exhaustion in lymphoid tissues have been described during prodromal measles in both humans and NHPs^16,17^. In vitro and in vivo studies showed that memory T cells are more susceptible to MV infection than naive T cells, due to higher expression of CD150^17,18^. This difference in susceptibility is less pronounced in the B cell lineage, with both naive and memory B cell subsets being highly susceptible and permissive to MV infection in vitro^17,19^. Based on these findings, we hypothesised that MV causes immunological amnesia by infecting and depleting pre-existing memory lymphocytes^17,20^. Consistent with this hypothesis, a subsequent epidemiological study found that rates of non-measles infectious disease mortality are tightly coupled to measles incidence—with a greater mortality rate at higher recent measles incidence. It was concluded that the reduction in host resistance following measles infection may extend over a period of more than 2 years^2^.

In the Netherlands, a monovalent measles vaccine was introduced into the national immunisation programme in 1976. Since 1987, measles vaccine has been offered as multivalent measles––mumps–rubella (MMR) vaccine to children at the age of 14 months and 9 years. Since then, nationwide MMR vaccination coverage has remained close to 95%^21^. However, large measles outbreaks among unvaccinated individuals who belong to socially and geographically clustered communities still occur occasionally^22,23^. In 2013, a measles outbreak occurred largely among the Orthodox Protestant community that refuses vaccination on religious grounds, with more than 2600 cases reported^23^. This outbreak presented us with a unique opportunity to study the pathogenesis of measles-associated immune suppression in natural measles patients. Here we show that MV viraemia is mediated by infected memory T cells and naive and memory B cells. In addition, we show that measles causes significant changes in the frequency of circulating lymphocyte subsets, which are still detectable more than a month after recovery.

## Results

### Characterisation of patients with early acute measles

**Fig. 1:**
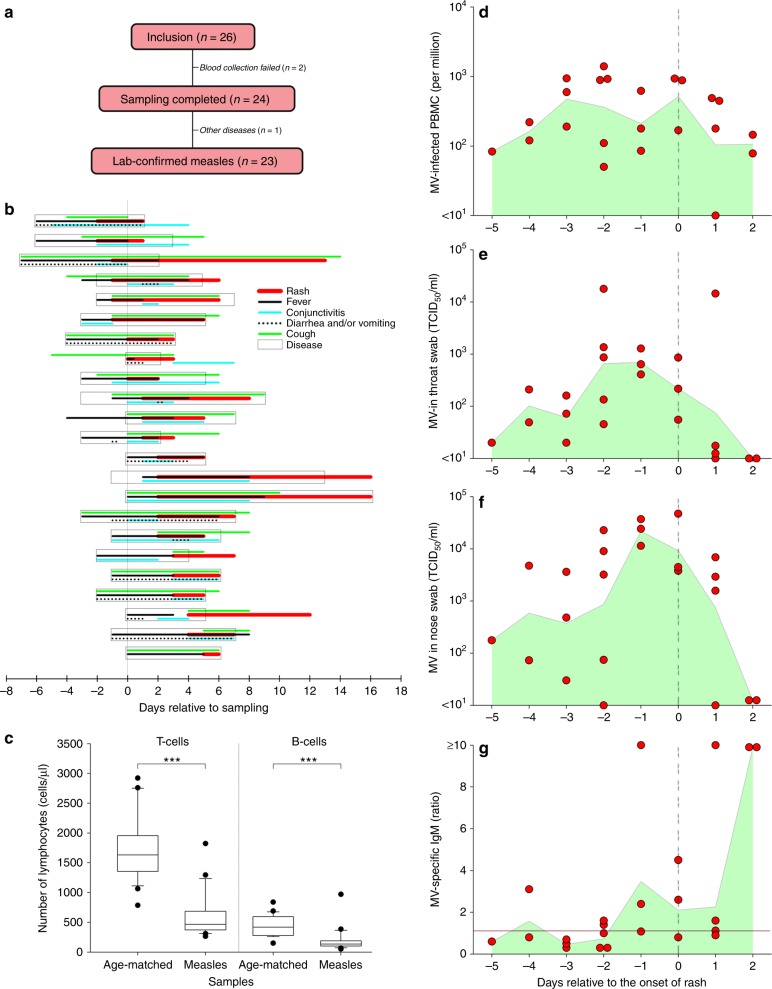
Cohort A patient information. **a** Number of inclusions, completed samples and laboratory-confirmed measles cases of Cohort A. **b** The onset and the duration of pathognomonic symptoms of measles experienced by each child as observed and reported by the parents. Red line: rash; black line: fever; cyan line: conjunctivitis; dotted line: diarrhoea and/or vomiting; green line: cough; box: disease. **c** The numbers of T and B cells per 1 µl of blood of Cohort A children in comparison with those of age-matched healthy children (*n* = 23). Statistical differences in the absolute numbers of T and B cells in the blood of acute measles patients in comparison to age-matched healthy donors were analysed by Mann–Whitney rank sum test. Centre lines of the box plots represent medians. Lower and upper boundaries of the boxes represent first and third quartiles, respectively. Lower and upper whiskers represent the 10th and 90th percentiles of the data, respectively. Dots represent outliers. **d** The number of infected cells per one million PBMC and **e**, **f** infectious MV isolated from throat and nose swabs relative to the onset of rash (*n* = 23 donors). Each red dot represents a unique donor. Green shaded area indicates geometric mean of the samples from each time point. **g** The presence of MV-specific IgM in plasma of each Cohort A child relative to the onset of rash (*n* = 23 donors). Each red dot represents a unique donor. The green shaded area indicates the mean of the samples from each time point, the red line indicates the lowest threshold (ratio 1.1) of detection for reactive MV-specific IgM as indicated by the manufacturer, and the vertical dashed line represents the onset of rash

**Fig. 2:**
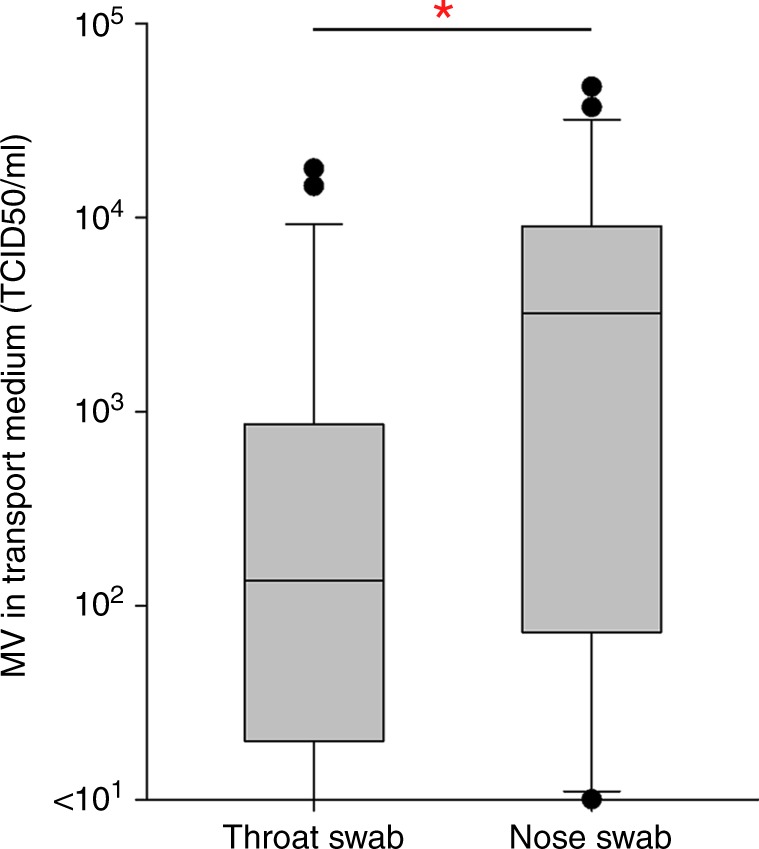
MV titres detected in transport medium of throat and nose swabs collected from early acute measles patients in Cohort A (*n* = 23). Centre lines of the box plots represent medians. Lower and upper boundaries of the boxes represent first and third quartiles, respectively. Lower and upper whiskers represent the 10th and 90th percentiles of the data, respectively. Dots represent outliers. Statistical analysis was performed using Mann–Whitney rank sum test. *P < 0.05

We performed an observational cohort study, and enrolled 26 unvaccinated children (ages ranged from 4 to 13 years old) with clinical signs of prodromal measles into Cohort A. A complete set of clinical specimens was successfully collected from 24 children, of which 23 children experienced laboratory-confirmed measles (Fig. 1a). Clinical signs as recorded by the parents included classical measles symptoms like fever, conjunctivitis, coughing, sneezing, throat pain and skin rash. More than half of the children were also reported to have diarrhoea and/or vomiting (Fig. 1b). Dizziness, ear pain, fatigue, nosebleed and mouth sores were also reported, albeit less frequent. In 17 out of 23 patients, specimens were collected during the prodromal stage of measles, i.e. before the onset of rash (Fig. 1b). Total numbers of T and B cells in peripheral blood were significantly decreased in the early acute measles patients compared to those of age-matched healthy controls, demonstrating measles-induced lymphopenia (Fig. 1c). MV infection causes cell-associated viraemia, and the virus was isolated from peripheral blood mononuclear cells (PBMC) obtained from 22 out of 23 patients (Fig. 1d). In addition, the virus was efficiently shed from the respiratory tract, as demonstrated by virus isolation from throat swabs of 20 out of 23 patients (Fig. 1e) and nose swabs of 21 out of 23 patients (Fig. 1f). Interestingly, virus titres detected in nose swabs were higher than those detected in throat swabs (*P* = 0.021) (Fig. 2), and virus shedding on average peaked between 2 days and 1 day before the onset of rash (Fig. 1e, f). In accordance with the fact that the samples were obtained during prodromal or early measles, the majority still contained undetectable or low levels of MV-specific IgM antibodies (Fig. 1g; Supplementary Fig. 1). From one of the patients sampled 1 day after onset of rash, we failed to isolate MV from either swabs or PBMC. In this patient, high MV-specific IgM (Fig. 1g) were detected, confirming the laboratory diagnosis (Supplementary Fig. 1 and Supplementary Data 1). This patient had already developed a neutralising antibody response (Supplementary Data 1, patient A01), explaining our inability to isolate virus from PBMC. Combined, these data provide an overview of the clinical and virological characteristics of the early stages of natural MV infection in unvaccinated children.

### MV tropism during viraemia

**Fig. 3:**
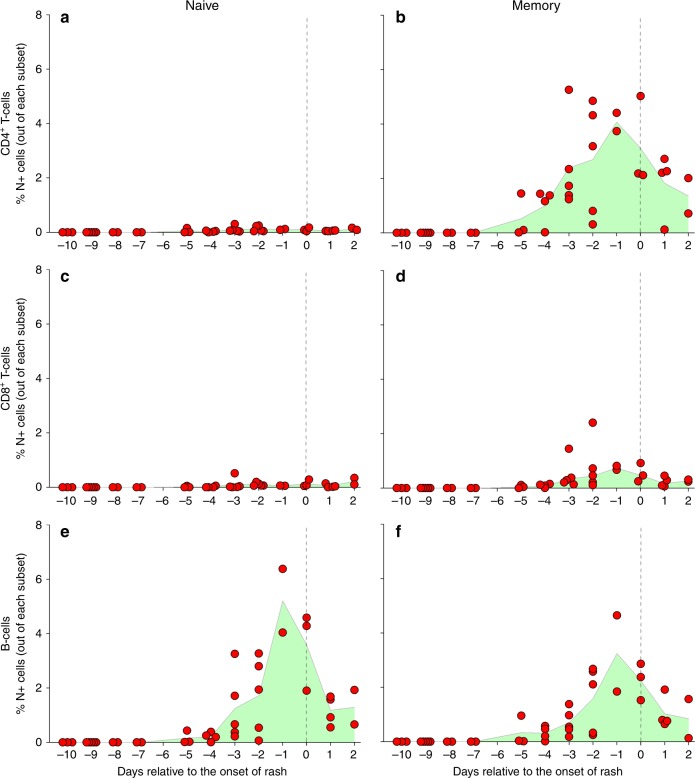
Frequencies of MV-infected lymphocytes during acute measles. **a**–**f** Frequencies of MV-N^+^ cells in naive and memory lymphocyte subsets relative to the onset of rash (*n* = 40 donors). Each red dot represents a unique donor, the green shaded area indicates the mean of the samples from each time point, and the vertical dashed line represents the onset of rash

To determine the phenotype of MV-infected cells in the PBMC collected from early acute measles patients in Cohort A, we performed surface staining of lymphocytes (Supplementary Table 1) and subsequent intracellular staining with an antibody specific to the MV nucleoprotein (MV-N), followed by flow cytometry. One PBMC sample from Cohort A did not contain enough viable cells to allow phenotypic identification. Additionally, in this analysis we included PBMC samples collected from 18 children initially enrolled into the second cohort of this study (Cohort B) that were later shown to be in the incubation phase of measles. MV-infected cells were defined as MV-N^+^ cells and expressed as a percentage out of their respective lymphocyte subset (Supplementary Fig. 2). MV-infected cells were hardly detected in the naive CD4^+^ and CD8^+^ T cell subsets (Fig. 3a, c). In contrast, MV-infected cells were detectable in the CD4^+^ and CD8^+^ memory T cell subsets (Fig. 3b, d), and in both the naive and memory B cell subsets (Fig. 3e, f). The percentages of MV-infected cells on average peaked 1 day before onset of rash.

### Prospective sampling of unvaccinated children

**Fig. 4:**
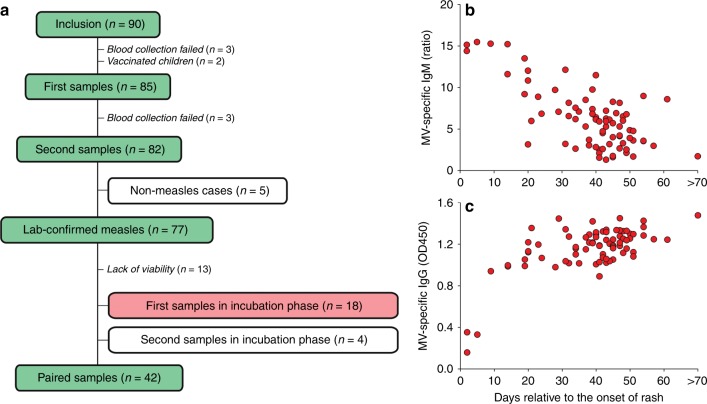
Cohort B patient information. **a** Number of inclusions and number of laboratory-confirmed measles and non-measles samples collected before and after the measles outbreak. White box indicates samples not included in measurement. Red box indicates samples included in the identification of MV-infected cells. **b** The levels of MV-specific IgM and **c** IgG in plasma of each Cohort B child relative to the onset of rash (*n* = 77 donors). Each red dot represents a unique donor

It was previously described that MV depletes lymphocytes in experimentally infected NHPs, as detected in peripheral blood by lymphopenia and in lymphoid tissues by follicular exhaustion^17^. In the current study, lymphopenia was confirmed in naturally infected measles patients (Fig. 1c), with MV preferentially infecting memory lymphocyte subsets (Fig. 3). To assess changes in the composition of lymphocyte subsets before and after measles, we enrolled 90 children without clinical signs into Cohort B (Fig. 4a). The first PBMC samples (further termed as pre-measles samples) were collected shortly before the start of the Dutch primary school holidays, which lasted for 6 weeks. Blood collection failed in three children and two other children turned out to be vaccinated for measles and thus their samples were excluded from the analyses. After the holidays, the second blood samples, further termed as post-measles samples, were collected from children who had experienced measles (Supplementary Fig. 3). Paired blood samples were successfully collected from 82 children, aged between 6 and 13 years old during the time of sampling. Some children (*n* = 5) never developed measles during the study period, as proven by the absence of MV-specific antibodies in their second blood sample (Supplementary Fig. 1f). The other 77 children experienced laboratory-confirmed measles during the study period, since all pre-measles samples were negative for MV-specific IgG and all post-measles samples contained MV-specific IgM and IgG (Fig. 4b, c, Supplementary Fig. 1 and Supplementary Data 2). Median time between pre-measles sample collection and parent-reported onset of rash was 16 days (range 3–98). Parents assessed the severity of the disease: 34 children were reported with mild and 43 with severe measles (Supplementary Data 2).

### Impact of measles on immune cell subsets

**Fig. 5:**
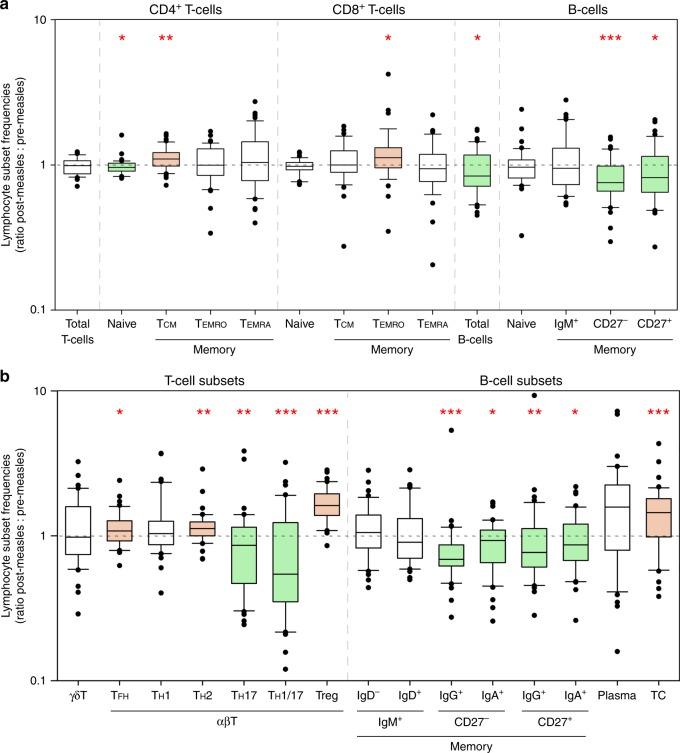
Significant changes in the frequencies of different lymphocyte subsets after measles. Frequency ratios of **a** naive or memory lymphocyte subsets or **b** functionally distinct T and B cell subsets (*n* = 42 paired samples). The ratio was calculated as the frequency of a subset after measles divided by the frequency of the same subset before measles. Horizontal dashed line indicates no changes (‘ratio = 1’) in frequency after measles. Ratio ‘>1’ indicates increase and ratio ‘<1’ indicates decrease in lymphocyte subset frequency after measles. Vertical dashed lines separate different lymphocyte subsets. Th1/17: Th1Th17 cells. CD27^+^IgM^+^IgD^−^ B cells are also known as IgM-only memory B cells. CD27^+^IgM^+^IgD^+^ B cells are also known as natural effector cells. TC: transitional B cells. Green box represents significant decrease. Orange box represents significant increase. Statistical differences in frequencies of lymphocyte subsets before and after measles were analysed by two-tailed paired *t*-test or Wilcoxon signed-rank test. Centre lines of the box plots represent medians. Lower and upper boundaries of the boxes represent first and third quartiles, respectively. Lower and upper whiskers represent the 10th and 90th percentiles of the data, respectively. Dots represent outliers. *P < 0.05; **P < 0.01; ***P ≤ 0.001

After exclusion of children who developed skin rash within 10 days after collection of the pre-measles samples (*n* = 18; these samples were used instead for phenotyping of MV-infected cells) or within 10 days before collection of the post-measles samples (*n* = 4), and elimination of samples that lacked viability (*n* = 13), 42 paired PBMC samples were available for the assessment of phenotypes and frequencies of lymphocyte subsets before and after measles. Total IgM, IgA and IgG levels in the pre- and post-measles plasma samples of these 42 donors were comparable (Supplementary Fig. 4). We used three separate surface marker-staining sets (Supplementary Table 1) and performed flow cytometry. As analysis by unsupervised learning^24^ showed segregation into clusters but did not allow identification of specific phenotypes (Supplementary Fig. 5), we proceeded with classical gating methods. Frequencies of specific T and B cell subsets were determined after gating for viable single cells as described earlier^19^. We expressed the changes in certain subsets as ratios of lymphocyte subset frequencies after and before measles. No changes were observed in the frequency of total circulating T cells after measles. However, there was a significant decrease in the frequency of CD4^+^ naive T cells, concomitant with an increase in the frequency of CD4^+^ Tcm cells. In the CD8^+^ T cell population, a significant increase was observed among the Temro cells. In the B cell population, there was a significant decrease in the frequency of total circulating B cells, mainly due to the loss of CD27^−^ and CD27^+^ immunoglobulin class-switched memory B cells after measles (Fig. 5a and Supplementary Fig. 6). Upon examination of frequencies of functionally distinct T cell subsets before and after measles, we found significant increases in the frequencies of follicular helper T (Tfh), helper T (Th)2, and regulatory T (Treg) cell subsets after measles. Furthermore, in accordance with the high susceptibility patterns to MV infection observed in vitro, Th17 and Th1Th17 cell frequencies were significantly decreased after natural measles^19^. We also assessed the frequencies of functionally distinct B cell subsets before and after measles and found significant losses of IgG^+^ and IgA^+^ memory B cells, accompanied by a significant increase in the frequencies of transitional B cells (Fig. 5b). Moreover, the changes in lymphocyte subset frequencies were overall more pronounced in children with severe than those with mild measles (Supplementary Figs. 7 and 8).

## Discussion

Measles is associated with immune suppression, leading to increased susceptibility to secondary infections. Immune suppression is not only the major driver of measles-associated mortality in developing countries, but may also explain the previously reported association between measles and non-measles infectious disease childhood mortality: an effect that extends over 2 years following the acute stage of the disease^2^. At the same time, measles also causes immune activation and induces lifelong immunity to measles. This apparent contrast is commonly referred to as the measles paradox. Although several potential underlying mechanisms have been described in literature, there is no consensus about the relative importance of these hypotheses. Based on observations from experimentally infected NHPs, we have recently proposed the immune amnesia model as a potential explanation for the mechanism underlying the measles paradox. In 2013, a large measles outbreak occurred among the Orthodox Protestant community in the Netherlands and provided a unique opportunity to study measles-associated immune suppression in naturally infected measles patients. We performed a prospective cohort study in unvaccinated children and successfully collected clinical samples before, during and after measles. MV-infected lymphocytes were detected in prodromal and early measles blood samples, and infectious virus was recovered from nose swabs, throat swabs and PBMC. We successfully identified the phenotypes of infected peripheral lymphocytes during measles and detected significant changes in lymphocyte composition after measles.

In Cohort A, parents reported the onset and duration of clinical symptoms, such as fever and skin rash. It should be emphasised that the reports have been recorded based on subjective observations of the parents and thus must be interpreted with caution. Interestingly, more children were reported with coughing than sneezing, and coughing was reported to occur several days before or on the day of the onset of rash in the majority of children. Coughing, potentially caused by measles-associated damage to the respiratory tract epithelium, likely contributes to the highly efficient airborne transmission of MV, since it allows MV particles to be expelled as infectious aerosols. During this time the highest infectious viral loads were detected in nose swabs. This suggests that MV replication in the nasopharynx rather than in the tracheobronchial airways contributes to the high infectiousness of measles^11,25–27^. Taking into consideration that the swab samples were diluted in transport medium, the actual concentration of infectious virus in the nasopharyngeal mucus must have been substantially higher than the titres assessed by our assays.

We were able to determine the phenotypes and frequencies of MV-infected circulating lymphocytes during prodromal measles. We found that, in the CD4^+^ and CD8^+^ T cell populations, memory cells were predominantly infected. In the B cell population, both naive and memory cells were infected. These findings are in agreement with previous in vitro observations and support our immune amnesia hypothesis^14,17,19^. Although the preferential infection of memory T cells by MV can be explained by the higher expression of the cellular receptor CD150 on memory T cells compared to naive T cells, this is not the case within the B cell populations. The expression of CD150 is a prerequisite and determines the susceptibility of lymphocytes to MV infection. However, the permissiveness to replicate and produce infectious progeny viruses likely depends on other intracellular factors, such as activation state or cellular metabolism. In this case, we have previously shown that B cells have higher permissiveness for MV than T cells^19^.

A recent study investigating the phenotype of in situ MV-infected cells in fatal measles cases identified macrophages and dendritic cells as predominant infected cells^28^. This observation fits well with pathological observations in experimentally infected NHPs, in which MV-infected myeloid cells were detected both early and late after measles. In contrast, MV-infected lymphocytes were detected at a high frequency both in peripheral blood and in lymphoid tissues for only a short period^14,17^. We speculate that MV infection of lymphocytes is more cytolytic than infection of myeloid cells. In this study, we did not detect MV-infected myeloid cells in peripheral blood. The only myeloid cell population present in substantial numbers in peripheral blood are monocytes, which do not express CD150 and are not susceptible to MV infection^14^. Dendritic cells, which proved to be susceptible to MV infection in NHPs and fatal measles cases^14,28^, are present in peripheral blood in low numbers which does not allow meaningful analysis of MV infection by flow cytometry. It is important to note that, in NHPs, the frequencies of MV-infected cells in lymphoid tissues were consistently higher than those detected in peripheral blood^14,17^. The similarity of both the frequencies and phenotypes of MV-infected cells in PBMC of natural measles patients to those previously observed in NHPs justifies the translation of these observations from animal model studies to natural measles in humans.

Analysis of the composition of T cell subsets before and after measles showed a decreased frequency of CD4^+^ naive T cells and increased frequencies of CD4^+^ Tcm and CD8^+^ Temro. Other memory T cell subsets showed both increased and decreased frequencies, without a consistent pattern. This observation fits well with our immune amnesia model, in which depletion of infected memory T cells is masked by the expansion of newly generated and phenotypically similar MV-specific and bystander T cells^17,20^. Similar to observations in experimentally infected NHPs, the balance between depletion and expansion may be expected to vary among donors. Since the changes in T cell subset frequency can be caused by antigen activation or homoeostatic turnover^2,17^, further investigations of the fate of pre-existing antigen-specific lymphocyte subsets after measles are required.

We further assessed the changes in the frequencies of functionally distinct T cell subsets. Although γδ T cells have been reported to expand in the presence of MV or MV-infected cells^29^, we did not observe any significant changes in this subset in our study. We observed a significant increase in the frequency of circulating CXCR5^+^ Tfh cells after measles. CXCR5 allows T cells to localise to B cell follicles and germinal centres to support B cell differentiation. However, since lymphocyte depletion and follicular exhaustion have been reported in the lymph nodes of experimentally infected NHPs, the increase of these circulating Tfh cells after measles is likely due to expansion of new migratory populations. Circulating Tfh and Th2 cells were the least susceptible among the functionally distinct T cell subsets to in vitro MV infection^19^. This low susceptibility might have allowed the cells to survive and expand during and following infection in vivo, thus providing support to B cells to differentiate into antibody-producing plasma cells. In contrast, circulating Th17 and Th1Th17 were the most susceptible subsets to in vitro MV infection and accordingly their frequencies decreased significantly after in vivo MV infection^19^. Treg cells are responsible for suppression of T cell responses after immune activation and elevated Treg cells have been observed in acute measles patients compared to healthy controls^30^. We showed that Treg cells were indeed increased in frequency after measles. However, whether this population remains high for a prolonged time and contributes to transient measles-induced immune suppression remains to be determined.

We found that peripheral B cells, especially those belonging to the class-switched memory subsets, were significantly reduced in frequency after measles. This loss was more prominent in the IgG^+^ memory B cells than in their IgA^+^ equivalents, most likely due to the higher susceptibility of IgG^+^ memory B cells to MV infection^19^. Accompanying the loss of memory B cells after measles was a significant influx of transitional B cells. Transitional B cells represent recent bone marrow emigrants in the circulation that will develop into mature naive B cells and have a reduced proliferation capacity^31^. We speculate that the expansion of this subset reflects a compensation for depletion of pre-existing memory B cells.

The mechanism underlying measles-induced immune suppression has never been truly understood. Altered cytokine profiles, bystander lymphocyte apoptosis and lymphocyte infection and depletion are a few of various proposed models to explain the mechanism^3,20^. However, available studies have not provided clear discrimination between the direct effect of MV infection on the lymphocyte population and the host responses to infection and lymphopenia after measles in vivo. In this study, we offer a new perspective on host responses to natural measles. MV infection causes lymphocyte depletion, with memory T and B cells being the most severely affected. Observations from bone marrow transplant recipients and HIV patients showed that newly generated T cells had a short life span due to spontaneous apoptosis, and these cells had a significantly decreased mitogenic activity^32–34^. Highly proliferating PBMC and apoptotic uninfected lymphocytes have been isolated from the blood of acute measles patients and reduced responsiveness to mitogen stimulation has been reported, suggesting that similar cells are present after measles^35,36^. B- and T cell receptor sequencing studies are required to determine whether the new emerging lymphocytes have similar antigen specificity to the lost ones.

Our study has a number of limitations. We observed a substantial level of biological variation in the changes in lymphocytes subset frequencies, with increases in some subjects while decreases were seen in others. We believe that our study contained a sufficient sample size to allow statistical analysis of the results and, despite individual variation, interpret changes as mediated by measles. Our study was limited to three time points (before, during and after measles). Although additional follow-up samples could have provided more insight in the longevity of measles-associated immune suppression, this would have increased the risk of non-compliance resulting in a loss of statistical power. Observational studies in healthy children are difficult, especially in terms of obtaining medical ethical clearance and informed consent and assent. Moreover, at the moment of preparation of our clinical study protocol, data suggesting that measles-associated immune suppression might continue for more than 2 years were not yet available. Thus, we did not see any reasons to justify additional sampling points. Another study limitation is the absence of baseline haematological and biochemical data for each sampling. This would have required collection of additional blood tubes, which we believe would have reduced the willingness of parents and children to participate in our study.

In conclusion, our study shows that measles viraemia in prodromal measles patients is mediated by MV-infected memory T cells and naive and memory B cells. We also show that measles has an impact on circulating lymphocyte subsets that lasts more than a month after recovery from the disease. These results offer an explanation for the observed long-term effects of measles on host resistance and underline the importance of maintaining measles vaccination coverage.

## Methods

### Ethical statement

Unvaccinated children aged 4 to 17 years old at the moment of sampling, without a history of natural measles, were recruited by distributing invitation letters and patient information forms via three Orthodox Protestant schools with low vaccination coverage (<20%) in the Netherlands. Children with chronic disease or immune suppression due to medication were excluded from participation. Despite the availability of free vaccination in the Dutch public healthcare system, the parents of our study participants refused all vaccinations on basis of religious grounds. They continued to do so in the face of an ongoing measles outbreak. No additional measures or interventions were available to prevent MV infections. Some patients developed complications, such as otitis media or pneumonia, and visited their general practitioners for treatment. However, none of the study participants required hospitalisation due to severe measles. An investigator and a research nurse visited the responding parents at home to provide them with additional explanation of the study. Clinical specimens were collected after verbal assent was obtained from participants younger than 12 years old or written informed consent from participants aged 12 years and older. Written informed consent from both parents was always obtained. The study protocol was approved by the medical ethical committee of Erasmus MC, the Netherlands (MEC-2013-302, CCMO register NL45323.078.13/2, see [http://www.toetsingonline.nl] and Supplementary Data 3).

### Clinical specimens

Children aged 4 to 13 years old (*n* = 26) in the prodromal phase or early stage of acute measles were enrolled into Cohort A. Complete sample sets were successfully collected from 24 children, of which 23 had laboratory-confirmed measles. Each sample set included throat and nose swabs (regular flocked swabs; COPAN Diagnostics Inc., USA) collected into 2.5 ml of virus transport medium and a single venous blood sample collected in a 10-ml heparin vacutainer tube^26^. Swab samples were homogenised using a vortex for 1 min, and viral transport medium aliquots were frozen at −80 °C. Cohort B included 90 children without clinical signs of measles, from whom 82 complete sample sets were successfully collected. Each sample set included paired blood samples collected in a 10-ml heparin vacutainer tube. A research nurse recorded parent-reported clinical signs and degrees of disease severity following telephone interviews. After laboratory tests, 77 children were confirmed to have measles between collection of the paired samples.

### Blood sample processing

In Cohort A, absolute counts of CD3^+^ T cells, CD19^+^ B cells and CD16^+^/CD56^+^ natural killer cells in whole-blood samples (50 µl) were determined by flow cytometry using Trucount tubes (BD Biosciences, USA)^37^. Identical data collected from age-matched healthy donors (*n* = 23)^38,39^ were included as controls. PBMC were isolated from heparinised blood samples of both cohorts. The tubes were centrifuged at 1200 *g* for 15 min followed by collection of plasma. Plasma was heat-inactivated for 30 min at 56 °C, and subsequently aliquots were frozen at −20 °C. Following centrifugation, cell pellets were reconstituted with phosphate-buffered saline (PBS) and subjected to density gradient centrifugation. PBMC were washed and frozen in RPMI-1640 medium (Lonza, Belgium) supplemented with 20% of foetal bovine serum (FBS) and 10% dimethyl sulfoxide at −135 °C.

### Cell culture and virus isolation

Vero cells expressing human CD150 (Vero-hCD150) were a kind gift from Prof Yusuke Yanagi, Kyushu University, Japan. The cells were grown in Dulbecco’s modified Eagle’s medium (Lonza) supplemented with 10% FBS, penicillin, streptomycin and l-glutamine (D10F medium)^40^. A human B-lymphoblastoid cell line (BLCL) was cultured in RPMI-1640 medium supplemented with 10% FBS, penicillin, streptomycin and l-glutamine (R10F medium)^41^. The cell line was generated in-house by Epstein-Barr virus-transformation of PBMC of a healthy adult donor. Human melanoma Mel-JuSo cells transfected with the full-length MV-Edmonston fusion (F) or hemagglutinin (H) genes (Mel-JuSo-F or Mel-JuSo-H)^42^ were grown in R10F medium. Virus isolations were performed by inoculation of Vero-hCD150 cells or BLCL with PBMC or virus transport medium from swab samples in two-fold serial dilutions (eight replicates per dilution). Viral cytopathic effects were monitored over a period of 3–7 days and a virus titre was calculated by determining the 50% endpoint using the formula of Reed and Muench^43^. One of the virus isolates (MVi/Dodewaard.NLD/29.13; genotype D8) was used for full genome sequencing as described previously (GenBank accession number: MG912592)^44^. This isolate is available via the European Virus Archive [https://www.european-virus-archive.com].

### MV-specific antibody detection

MV-specific IgM was detected using the Measles IgM capture EIA kit (Microimmune, UK). Results are expressed as a ratio of the optical density of the test sample over that of a reference sample (cut-off for positivity is 1.1). MV-specific IgG was detected using an in-house indirect MV IgG enzyme-linked immunosorbent assay^45^. Briefly, high-binding 96-well flat-bottomed plates were coated with beta-propiolactone-inactivated MV antigen (strain Edmonston) in PBS, and incubated with plasma diluted 1:300 in buffer containing PBS, 0.05% Tween-20, 5% milk and 1% rabbit serum. Specific antibodies were detected using horseradish peroxidase-labelled rabbit-anti-human IgG antibody (1:4,800; DAKO, Denmark, cat. no. P0214) and tetramethylbenzidine as a substrate. Results are shown as extinction at 450 nm with a reference filter of 620 nm. Each sample was measured in duplicate. MV F and H protein-specific IgM and IgG antibody levels were detected by flow cytometry^42^. Briefly, trypsinised cells were incubated with plasma samples diluted 1:100 in PBS supplemented with 3% FBS, and bound antibodies were detected using a FITC-labelled polyclonal rabbit-anti-human IgG conjugate (1:50; DAKO, cat. no. F0315). Fluorescence was measured on a BD FACS Canto II flow cytometer, and results are shown as geometric mean fluorescence expressed in arbitrary fluorescence units (AFU). Virus neutralising antibodies were detected using a fluorescent focus reduction neutralisation assay^45^. Briefly, Vero-hCD150 cells were seeded in a 96-well flat-bottomed plate in R10F medium 4 days prior to the experiment. Serial dilutions (2^2^–2^9^, each dilution was tested in duplicate) of the heat-inactivated plasma samples were prepared with recombinant MV strain Edmonston expressing EGFP (1:1) and incubated for 1.5 h at 37 °C. The plasma-virus mixture was then transferred to the 96-well plate containing Vero-hCD150 cells. After incubation for 4 h at 37 °C, fusion inhibitory protein (Bachem, Heidelberg, Germany) was added to each well to a final concentration of 0.2 mM to prevent cell-to-cell fusion. The cells were incubated at 37 °C for another 2 days, after which they were washed with PBS and fixed with 2% paraformaldehyde in PBS. Single fluorescent cells were counted automatically using an ImmunoSpot S6 analyser (CTL, Bonn, Germany) and the 50% focus reduction titre was determined. Results were expressed in international units per ml using the WHO third international reference serum for measles (NIBSC, South Mimms, United Kingdom).

### Flow cytometry

Frequencies of infected cells and lymphocyte subsets in PBMC were determined by flow cytometry using a Fortessa Cell Analyser (BD Biosciences). PBMC were thawed on the day of measurement and washed twice with Iscove’s Modified Dulbecco’s Medium (IMDM; Lonza) supplemented with 10% FBS (I10F medium). The cells were incubated in I10F medium supplemented with 50 U/ml benzonase (Merck Millipore, USA) for 30 min prior to centrifugation and suspension in PBS with antibodies for flow cytometry. In Cohort A, an multicolour staining was performed to identify MV-infected lymphocyte subsets (see Supplementary Table 1)^19^. Viable cells were identified using LIVE/DEAD Fixable Dead Cell Stain kit with Aqua fluorescent reactive dye (Life Technologies, USA). The presence of MV MV-N in the cells was detected by intracellular staining with an MV-N-specific monoclonal antibody conjugated with FITC (clone 83KKII; Merck Millipore), using a fixation and permeabilisation kit according to the manufacturer’s instructions (BD Biosciences)^19^. Antibody dilutions and catalogue numbers used in this study are available in Supplementary Table 1. MV infection levels within each lymphocyte subset were assessed by detection of cells expressing MV-N and shown as percentages. In Cohort B, three separate multicolour stainings were performed to identify T and B cell subsets, as specified before (Supplementary Table 1)^19,37,46^. Viable cells were identified using Fixable Viability Stain 520 (BD Biosciences). All gating strategies used for analyses in this study are shown in Supplementary Figs. 9–12.

### Statistical analyses

The statistical differences in the absolute number of T and B cells in the blood of acute measles patients in comparison to age-matched healthy donors and the statistical differences in virus titres of throat and nose swabs were analysed by Mann–Whitney rank sum test. The ratio of lymphocyte subset frequency before and after measles was calculated as the frequency of the subset after measles divided by the frequency of the same subset before measles. Ratio ‘1’ indicates no changes, while ratio ‘>1’ indicates increase and ratio ‘<1’ indicates decrease in lymphocyte subset frequency after measles. Statistical differences in frequencies of lymphocyte subsets before and after measles were analysed by two-tailed paired *t*-test or Wilcoxon signed-rank test. *P < 0.05, **P < 0.01, ***P ≤ 0.001. Each measurement in this study was performed once, unless mentioned otherwise.

### Code availability

Software used for unsupervised analysis of flow cytometry data (Supplementary Fig. 5), including R base packages and Rphenograph, have been previously published^24^. Linking scripts used to process the data have no access restrictions and are available upon request.

### Reporting summary

Further information on research design is available in the Nature Research Reporting Summary linked to this article.

## Electronic supplementary material

Supplementary Information

Description of Additional Supplementary Files

Supplementary Data 1

Supplementary Data 2

Supplementary Data 3

Reporting Summary

## Data Availability

All authors declare that all data generated and analysed in this study are included in this published article and its Supplementary Information files.

## References

[1] Rota PA (2016). Measles. Nat. Rev. Dis. Primers.

[2] Mina MJ, Metcalf CJ, de Swart RL, Osterhaus AD, Grenfell BT (2015). Long-term measles-induced immunomodulation increases overall childhood infectious disease mortality. Science.

[3] Griffin DE (2010). Measles virus-induced suppression of immune responses. Immunol. Rev..

[4] Gadroen Kartini, Dodd Caitlin N, Masclee Gwen M C, de Ridder Maria A J, Weibel Daniel, Mina Michael J, Grenfell Bryan T, Sturkenboom Miriam C J M, van de Vijver David A M C, de Swart Rik L (2018). Impact and longevity of measles-associated immune suppression: a matched cohort study using data from the THIN general practice database in the UK. BMJ Open.

[5] Moss WJ (2017). Measles. Lancet.

[6] WHO.. (2017). Progress towards regional measles elimination-worldwide, 2000–2016. Wkly Epidemiol. Rec..

[7] Kmietowicz Z (2018). “Tragedy” of 35 deaths from measles in Europe last year is unacceptable, says WHO. BMJ.

[8] Goodson James L., Alexander James P., Linkins Robert W., Orenstein Walter A. (2017). Measles and rubella elimination: learning from polio eradication and moving forward with a diagonal approach. Expert Review of Vaccines.

[9] Tatsuo H, Ono N, Tanaka K, Yanagi Y (2000). SLAM (CDw150) is a cellular receptor for measles virus. Nature.

[10] Noyce RS (2011). Tumor cell marker PVRL4 (nectin 4) is an epithelial cell receptor for measles virus. PLoS Pathog..

[11] Muhlebach MD (2011). Adherens junction protein nectin-4 is the epithelial receptor for measles virus. Nature.

[12] Lemon K (2011). Early target cells of measles virus after aerosol infection of non-human primates. PLoS Pathog..

[13] Mesman AW (2012). A prominent role for DC-SIGN+ dendritic cells in initiation and dissemination of measles virus infection in non-human primates. PLoS ONE.

[14] de Swart RL (2007). Predominant infection of CD150+ lymphocytes and dendritic cells during measles virus infection of macaques. PLoS Pathog..

[15] Laksono BM, de Vries RD, McQuaid S, Duprex WP, de Swart RL (2016). Measles virus host invasion and athogenesis. Viruses.

[16] Warthin AS (1931). Occurrence of numerous large giant cells in the tonsils and pharyngeal mucosa in the prodromal stage of measles. Arch. Pathol..

[17] de Vries RD (2012). Measles immune suppression: lessons from the macaque model. PLoS Pathog..

[18] Condack C, Grivel JC, Devaux P, Margolis L, Cattaneo R (2007). Measles virus vaccine attenuation: suboptimal infection of lymphatic tissue and tropism alteration. J. Infect. Dis..

[19] Laksono Brigitta M., Grosserichter-Wagener Christina, de Vries Rory D., Langeveld Simone A. G., Brem Maarten D., van Dongen Jacques J. M., Katsikis Peter D., Koopmans Marion P. G., van Zelm Menno C., de Swart Rik L. (2018). In Vitro Measles Virus Infection of Human Lymphocyte Subsets Demonstrates High Susceptibility and Permissiveness of both Naive and Memory B Cells. Journal of Virology.

[20] de Vries RD, de Swart RL (2014). Measles immune suppression: functional impairment or numbers game?. PLoS Pathog..

[21] van Wijhe M, McDonald SA, de Melker HE, Postma MJ, Wallinga J (2016). Effect of vaccination programmes on mortality burden among children and young adults in the Netherlands during the 20th century: a historical analysis. Lancet Infect. Dis..

[22] van den Hof S, Conyn-van Spaendonck MA, van Steenbergen JE (2002). Measles epidemic in the Netherlands, 1999-2000. J. Infect. Dis..

[23] Woudenberg T (2017). Large measles epidemic in the Netherlands, May 2013 to March 2014: changing epidemiology. Euro Surveill.

[24] Levine JH (2015). Data-driven phenotypic dissection of AML reveals progenitor-like cells that correlate with prognosis. Cell.

[25] Frenzke M (2013). Nectin-4-dependent measles virus spread to the cynomolgus monkey tracheal epithelium: role of infected immune cells infiltrating the lamina propria. J. Virol.

[26] Ludlow M (2013). Infection of lymphoid tissues in the macaque upper respiratory tract contributes to the emergence of transmissible measles virus. J. Gen. Virol..

[27] Ludlow M, McQuaid S, Milner D, de Swart RL, Duprex WP (2015). Pathological consequences of systemic measles virus infection. J. P athol.

[28] Allen, I. V. et al. Macrophages and dendritic cells are the predominant cells infected in measles in humans. *mSphere* **3**, pii: e00570-17 (2018). 10.1128/mSphere.00570-17 PMC5956143 29743202

[29] Bieback K, Breer C, Nanan R, ter Meulen V, Schneider-Schaulies S (2003). Expansion of human gamma/delta T cells in vitro is differentially regulated by the measles virus glycoproteins. J. Gen. Virol..

[30] Yu XL (2008). Measles virus infection in adults induces production of IL-10 and is associated with increased CD4+ CD25+ regulatory T cells. J. Immunol..

[31] Blair PA (2010). CD19(+)CD24(hi)CD38(hi) B cells exhibit regulatory capacity in healthy individuals but are functionally impaired in systemic Lupus Erythematosus patients. Immunity.

[32] Donnenberg, A. D., Margolick, J. B. & Donnenberg, V. S. in *Corticotropin-Releasing Factor and Cytokines: Role in the Stress Response*. Vol. 697, 770 (eds. Tache, Y. & Rivier, C.) 213–226 (New York Academy of Sciences, New York, 1995).

[33] Talmadge JE, Singh R, Ino K, Ageitos A, Buyukberber S (2000). Mechanisms of immune dysfunction in stem cell transplantation. Int. J. Immunopharmacol..

[34] Katsikis PD, Wunderlich ES, Smith CA, Herzenberg LA, Herzenberg LA (1995). Fas antigen stimulation induces marked apoptosis of T lymphocytes in human immunodeficiency virus-infected individuals. J. Exp. Med..

[35] Okada H (2000). Extensive lymphopenia due to apoptosis of uninfected lymphocytes in acute measles patients. Arch. Virol..

[36] Ward BJ, Johnson RT, Vaisberg A, Jauregui E, Griffin DE (1990). Spontaneous proliferation of peripheral mononuclear cells in natural measles virus infection: identification of dividing cells and correlation with mitogen responsiveness. Clin. Immunol. Immunopathol.

[37] Heeringa JJ (2018). Expansion of blood IgG4(+) B, TH2, and regulatory T cells in patients with IgG4-related disease. J. Allergy Clin. Immunol..

[38] Driessen GJ (2013). Common variable immunodeficiency and idiopathic primary hypogammaglobulinemia: two different conditions within the same disease spectrum. Haematologica.

[39] van den Heuvel D (2015). Persistent subclinical immune defects in HIV-1-infected children treated with antiretroviral therapy. AIDS.

[40] Ono N (2001). Measles viruses on throat swabs from measles patients use signaling lymphocytic activation molecule (CDw150) but not CD46 as a cellular receptor. J. Virol..

[41] van Binnendijk RS, van der Heijden RW, van Amerongen G, UytdeHaag FG, Osterhaus AD (1994). Viral replication and development of specific immunity in macaques after infection with different measles virus strains. J. Infect. Dis..

[42] de Swart RL, Vos HW, UytdeHaag FGCM, Osterhaus ADME, van Binnendijk RS (1998). Measles virus fusion protein- and hemagglutinin-transfected cell lines are a sensitive tool for the detection of specific antibodies by a FACS-measure immunofluorescence assay. J. Virol. Methods.

[43] Reed LJ, Muench H (1938). A simple method of estimating fifty percent endpoints. Am. J. Hyg..

[44] Phan MVT (2018). Complete genome sequences of six measles virus strains. Genome Announc..

[45] de Swart RL (2017). Needle-free delivery of measles virus vaccine to the lower respiratory tract of non-human primates elicits optimal immunity and protection. NPJ Vaccine.

[46] Nodland SE (2011). IL-7R expression and IL-7 signaling confer a distinct phenotype on developing human B-lineage cells. Blood.

