# Supplementary material for: Studies into the mechanism of measles-associated immune suppression during a measles outbreak in the Netherlands
Source: Nat Commun. 2018 Nov 23;9:4944. doi: 10.1038/s41467-018-07515-0 (PMC6251901; doi:10.1038/s41467-018-07515-0)
Supplement: Supplementary file 1 — Supplementary Information [file 41467_2018_7515_MOESM1_ESM.docx]

**Supplementary data**

**Supplementary Figure 1.** MV fusion protein- and haemagglutinin-specific IgM and IgG responses.

**Supplementary Figure 2.** Gating strategy to determine MV-infected cells, defined as MV-N^+^ cells and assessed by flow cytometry.

**Supplementary Figure 3.** Overview of sampling time points of Cohort B.

**Supplementary Figure 4.** Concentration of total immunoglobulins before and after measles.

**Supplementary Figure 5.** Comparison of lymphocyte subset frequencies found before and after measles based on unsupervised analyses.

**Supplementary Figure 6.** Comparison of lymphocyte subset frequencies before and after measles.

**Supplementary Figure 7.** Comparison of lymphocyte subset frequencies before and after measles in parent-reported mild measles cases.

**Supplementary Figure 8.** Comparison of lymphocyte subset frequencies before and after measles in parent-reported severe measles cases.

**Supplementary Figure 9.** Gating strategy to determine naive and memory lymphocyte subsets in Cohort A samples.

**Supplementary Figure 10.** Gating strategy to determine B-cell subsets in Cohort B samples.

**Supplementary Figure 11.** Gating strategy to determine T-cell subsets in Cohort B samples.

**Supplementary Figure 12.** Gating strategy to determine Th-cell subsets in Cohort B samples.

**Supplementary Table 1.** List of antibodies used in this study.


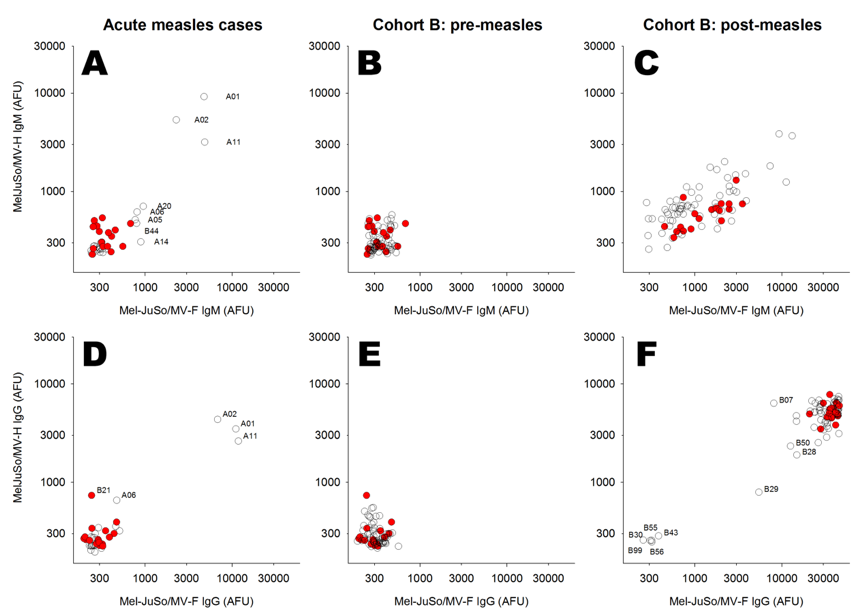


**Supplementary Figure 1: MV fusion (F) protein- and haemagglutinin (H) protein-specific IgM (A-C) and IgG (D-E).** Relationship between MV F protein- and H protein-specific IgM and IgG of (A and D) acute measles cases (n = 41 donors), (B and E) Cohort B pre- and (C and F) post-measles samples (n = 77 paired samples). Code represents patient ID (see Supplementary C and G). Each symbol represents one donor. Red circles represent the 18 donors from Cohort B whose pre-measles samples were found to be in the incubation phase of MV infection and were used in the panel of early acute measles patients. Results were obtained by flow cytometry using transfected Mel-JuSo cells (see reference 39), and are expressed in AFU (Arbitrary Fluorescence Units).


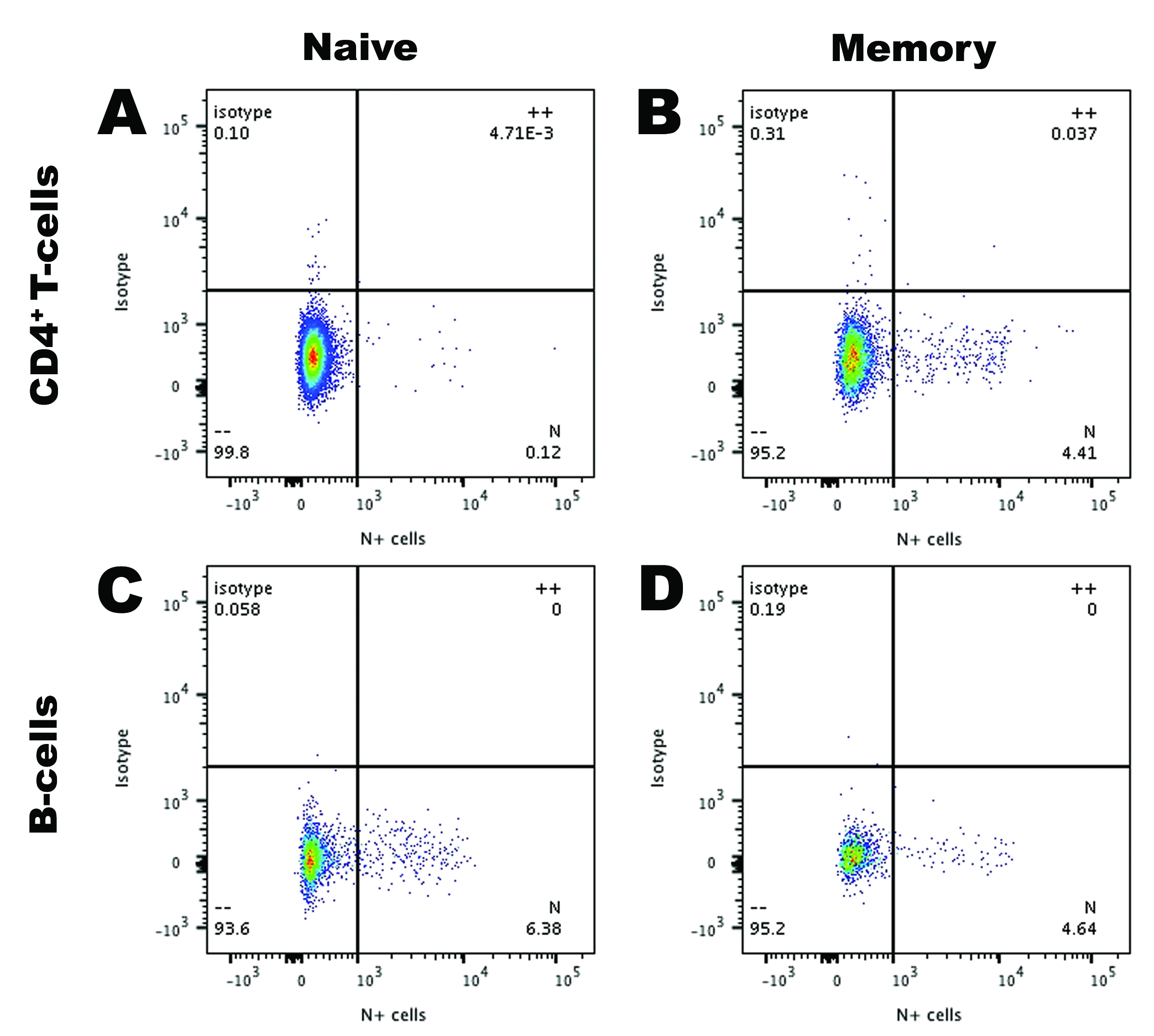


**Supplementary Figure 2: Gating strategy to determine MV-infected cells, defined as MV-N^+^ cells and assessed by flow cytometry.** (A) Percentages of N^-^ and (B) N^+^ CD4^+^ T-and (C-D) B-cells from one representative measurement, as shown in flow cytometry dot plots. Isotype control was included in the measurement to assess the level of background staining. Figures were chosen from one representative measurement.

**
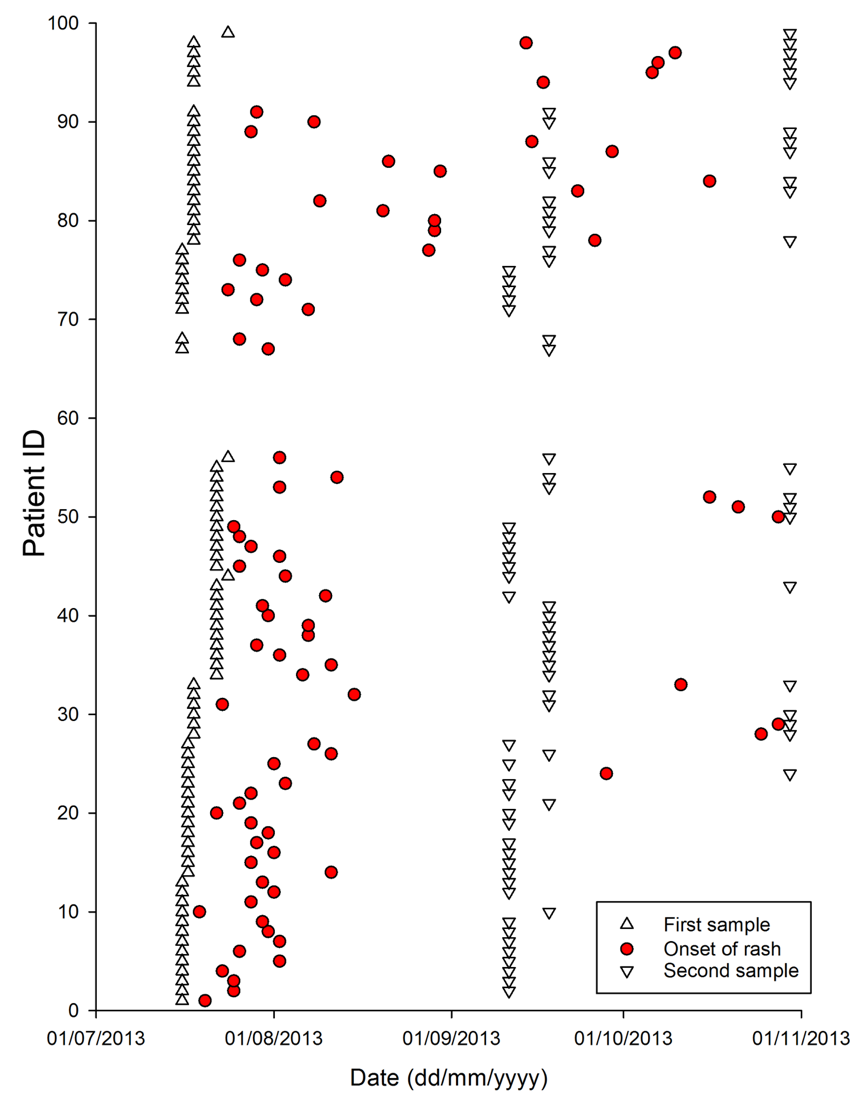
**

**Supplementary Figure 3: Overview of sampling time points of Cohort B.** The date of first sample collection (triangle), the date of the onset of rash (red circle) and the date of the second sample collection (inverted triangle) of all children included in Cohort B (n = 90).

**
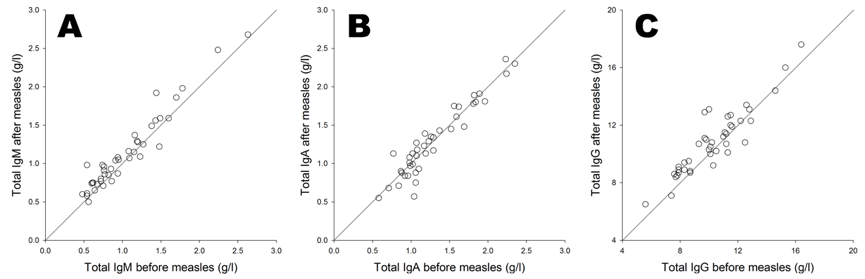
**

**Supplementary Figure 4: Concentration of total immunoglobulins before and after measles (n = 42 donors).** Relationships between (A) total IgM, (B) IgA and (C) IgG before and after measles. Each symbol represents one donor.


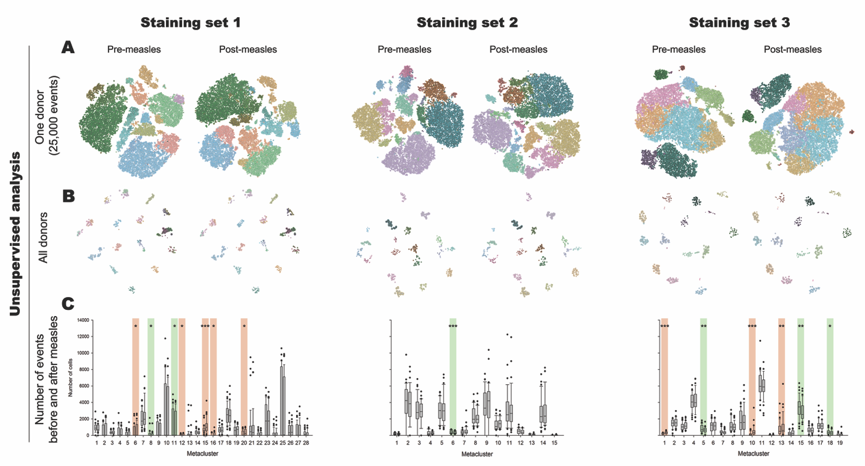


**Supplementary Figure 5: Comparison of lymphocyte subset frequencies found before and after measles based on unsupervised analyses.** Flow cytometry results of Cohort B samples were analysed using the unsupervised clustering method Phenograph to provide objective and unbiased separation of phenotypically distinct cell events. This analysis was performed in R using the Rphenograph package (Levine JH, *et al*. Data-Driven Phenotypic Dissection of AML Reveals Progenitor-like Cells that Correlate with Prognosis. Cell 162, 184-197 (2015)). For each sample separately, 25,000 viable single-cell events were selected to be able to compare cluster sizes between samples. These single cell events clustered using Phenograph, and the results were visualised on in two-dimensional t-SNE maps. Representative cells located in the centre of the detected clusters (the cluster centroids, further termed as “centromeres”) were selected from each sample and clustered using Phenograph to generate “metaclusters” that represent cell populations that were present in multiple samples. The primary clustering was performed with k = 12, 15, and 20 in set 1, 2, and 3, respectively, and the metaclustering was performed using k = 30 in all cases. To analyse the output of the Phenograph method, we used the R linear modelling (lm) functionality. Cell counts per sample per metacluster were analysed as sqrt(number.of.cells) ~ cluster.size + FACS.analysis.batch + patient + before_after.infection.  R citation:

@Manual{,

title = {R: A Language and Environment for Statistical

Computing},

author = {{R Core Team}},

organization = {R Foundation for Statistical Computing},

address = {Vienna, Austria},

year = 2018,

url = {https://www.R-project.org}

}


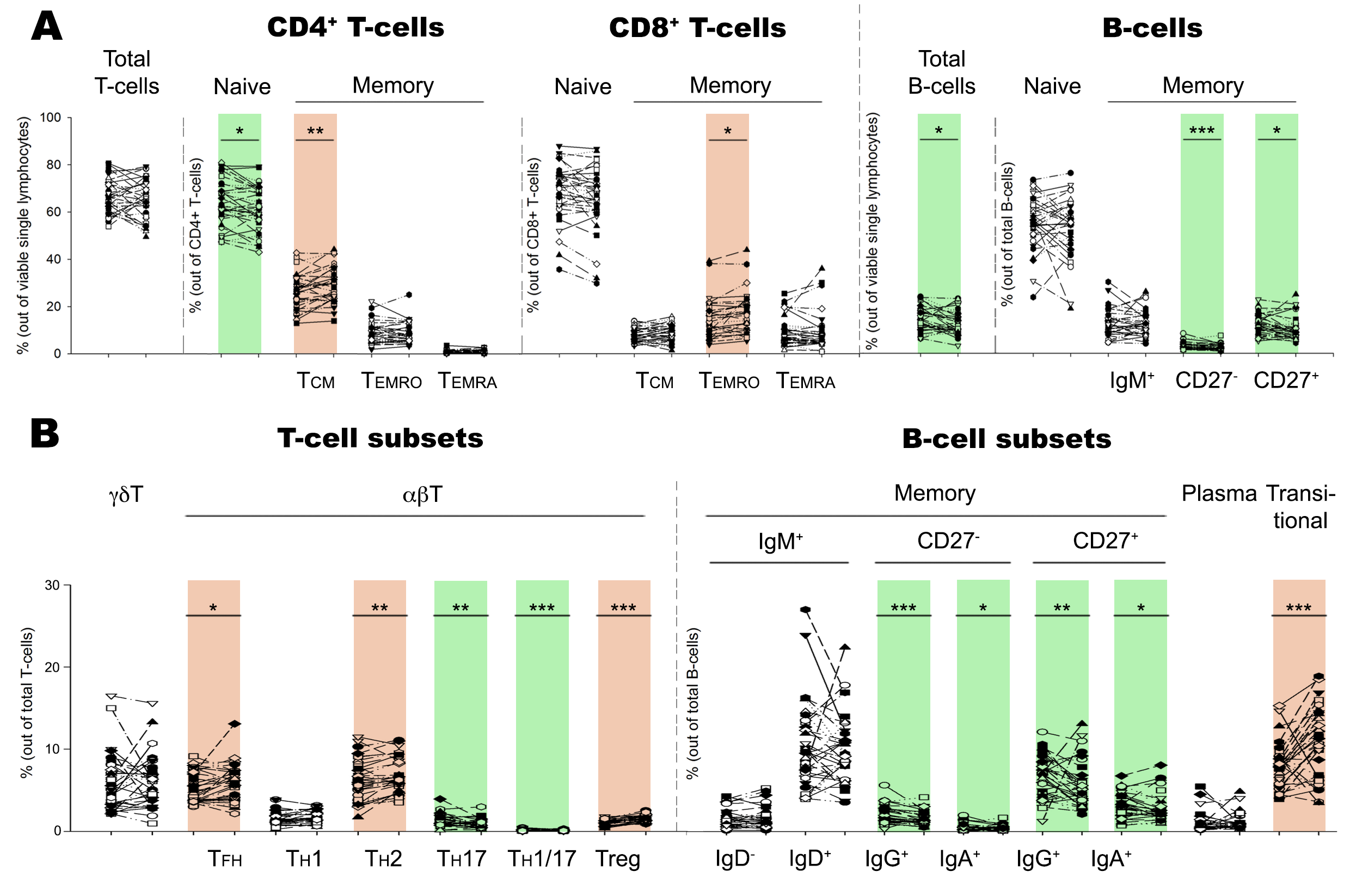


**Supplementary Figure 6: Comparison of lymphocyte subset frequencies before and after measles.** (A) Percentages of naive and memory and (B) functionally-distinct lymphocyte subsets before and after measles (n = 42 paired samples). Th1/17: Th1Th17 cells. CD27^+^IgM^+^IgD^-^ B-cells are also known as IgM-only memory B-cells. CD27^+^IgM^+^IgD^+^ B-cells are also known as natural effector cells. Green box represents significant decrease. Orange box represents significant increase. Statistical differences in frequencies of lymphocyte subsets before and after measles were analysed by two-tailed paired t-test or Wilcoxon signed-rank test. *: P < 0.05; **: P < 0.01; ***: P ≤ 0.001.


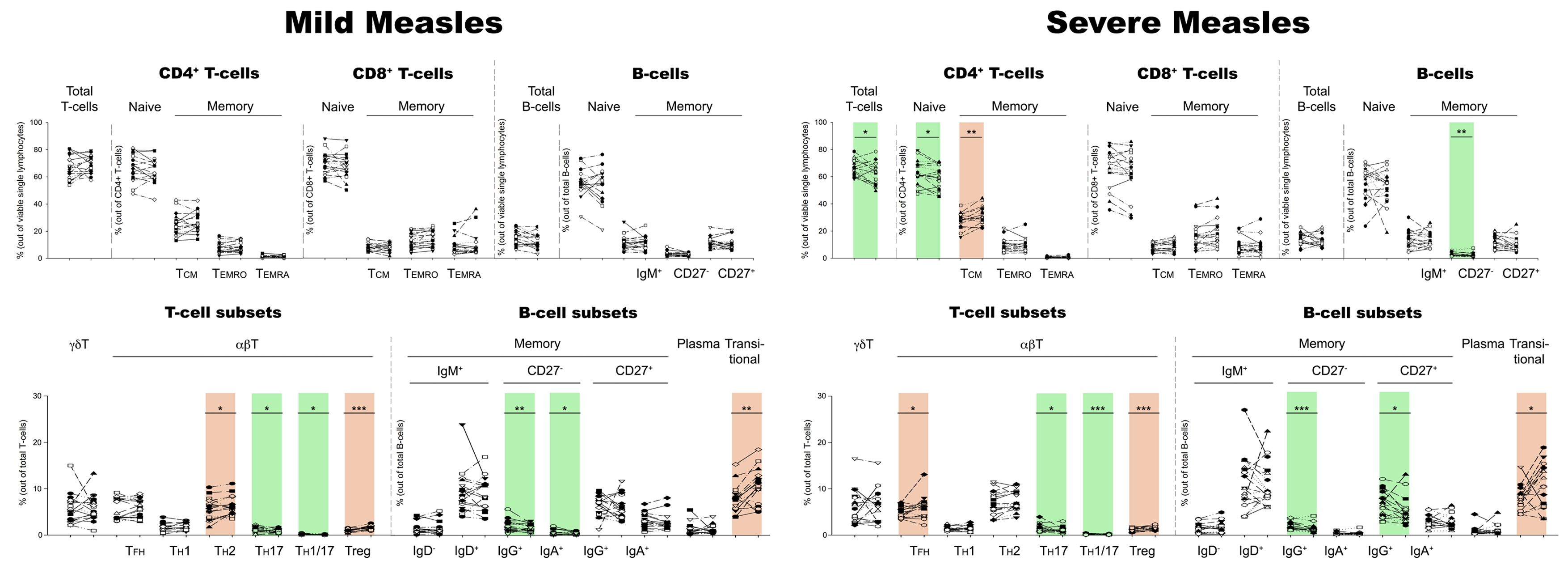


**Supplementary Figure 7: Comparison of lymphocyte subset frequencies before and after measles in parent-reported mild measles cases.** (A) Percentages of naive and memory and (B) functionally-distinct lymphocyte subsets before and after mild measles (n = 18 paired samples). Th1/17: Th1Th17 cells. CD27^+^IgM^+^IgD^-^ B-cells are also known as IgM-only memory B-cells. CD27^+^IgM^+^IgD^+^ B-cells are also known as natural effector cells. Green box represents significant decrease. Orange box represents significant increase. Statistical differences in frequencies of lymphocyte subsets before and after measles were analysed by two-tailed paired t-test or Wilcoxon signed-rank test. *: P < 0.05; **: P < 0.01; ***: P ≤ 0.001.

**
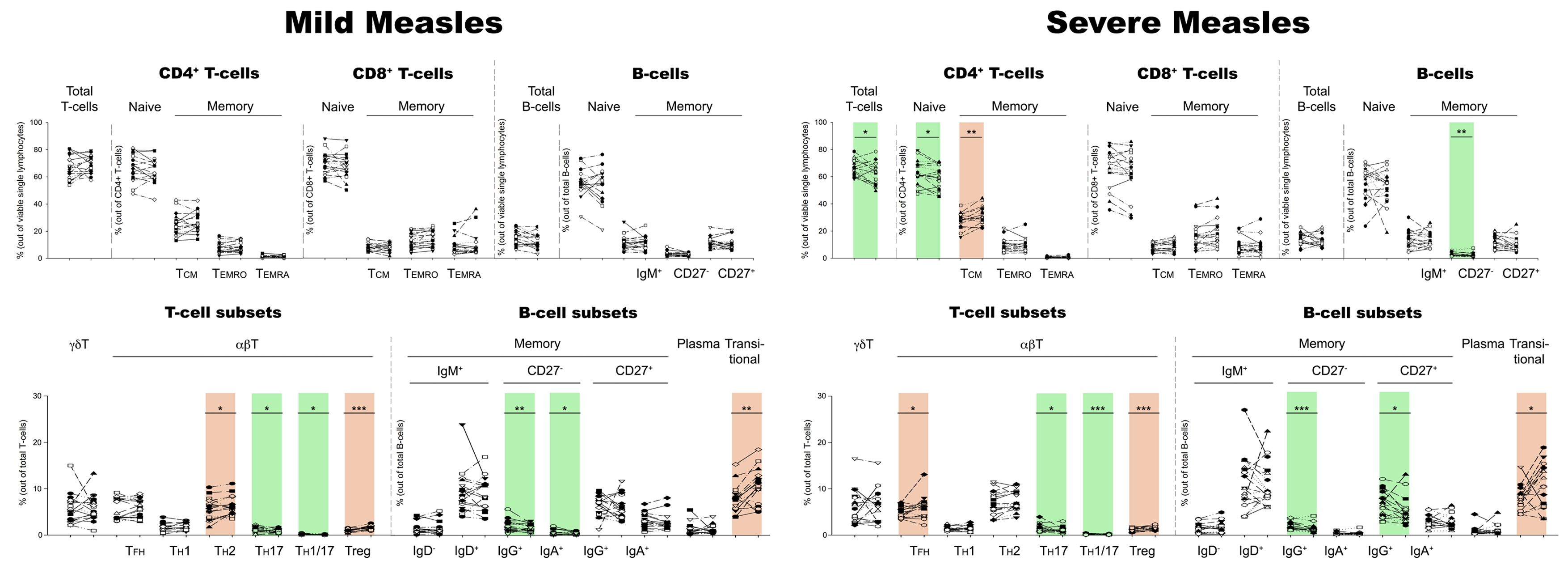
**

**Supplementary Figure 8: Comparison of lymphocyte subset frequencies before and after measles in parent-reported severe measles cases.** (A) Percentages of naive and memory and (B) functionally-distinct lymphocyte subsets before and after severe measles (n = 24 paired samples). Th1/17: Th1Th17 cells. CD27^+^IgM^+^IgD^-^ B-cells are also known as IgM-only memory B-cells. CD27^+^IgM^+^IgD^+^ B-cells are also known as natural effector cells. Green box represents significant decrease. Orange box represents significant increase. Statistical differences in frequencies of lymphocyte subsets before and after measles were analysed by two-tailed paired t-test or Wilcoxon signed-rank test. *: P < 0.05; **: P < 0.01; ***: P ≤ 0.001.


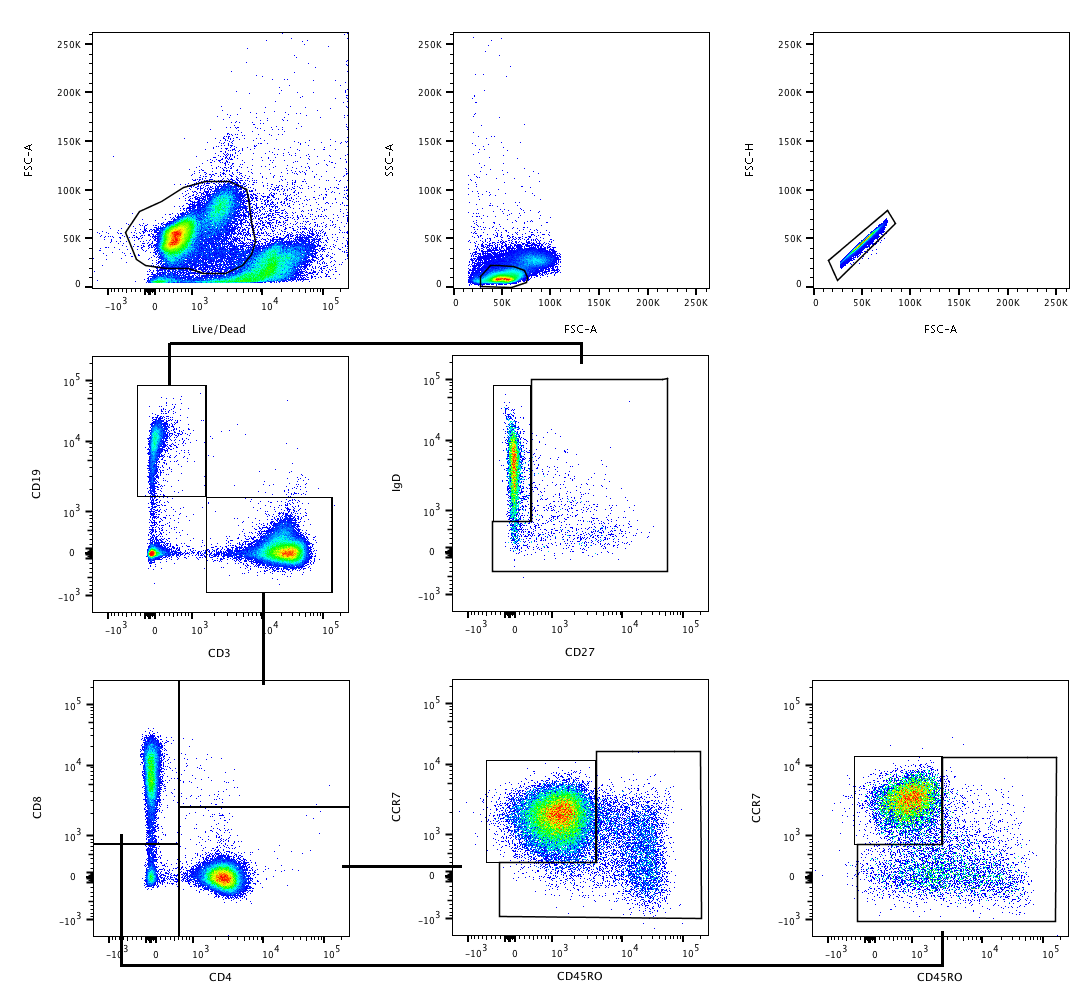


**Supplementary Figure 9: Gating strategy to determine naive and memory lymphocyte subsets in Cohort A samples.**

**
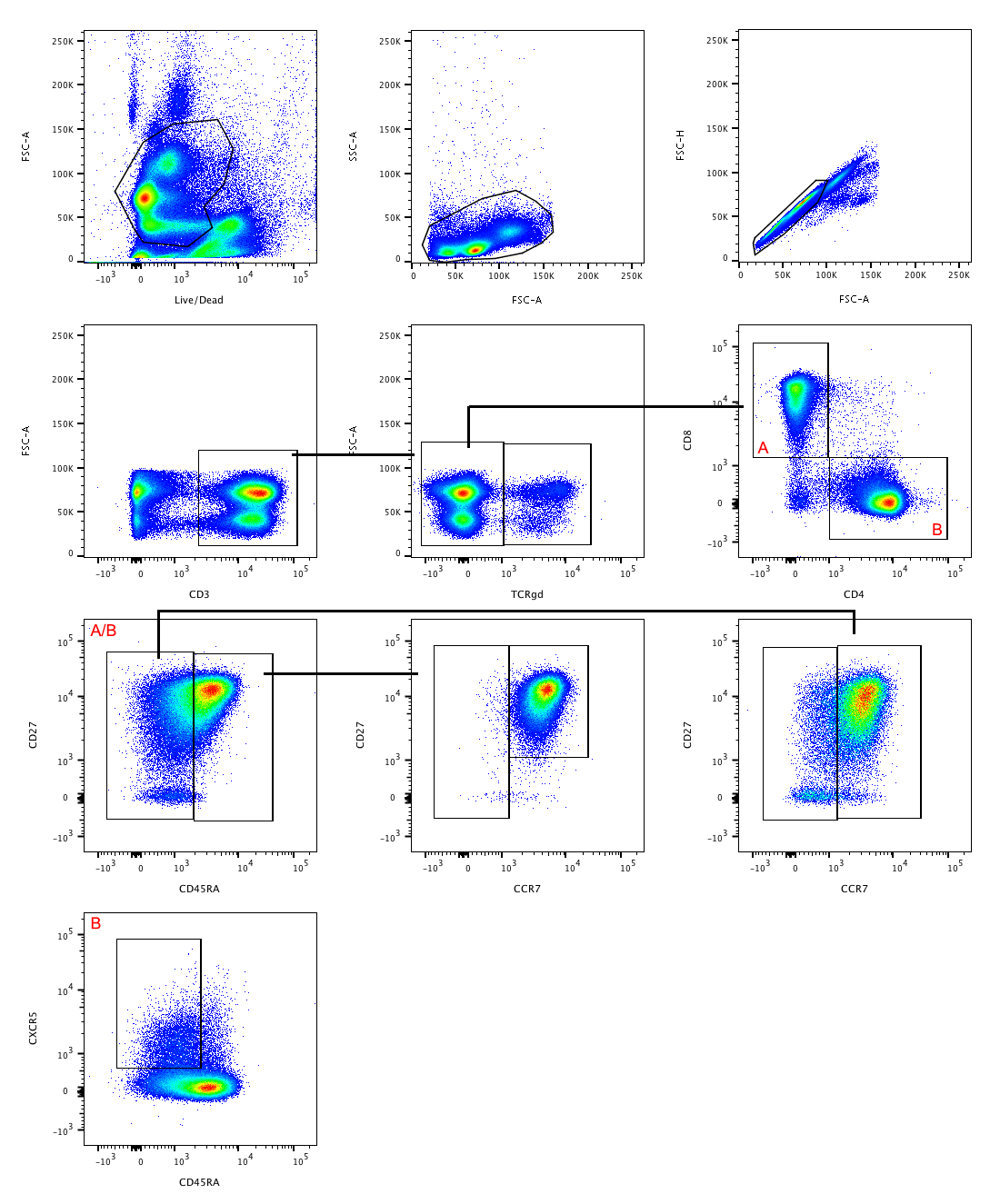
**

**Supplementary Figure 10: Gating strategy to determine T-cell subsets in Cohort B samples.** TCRgd: γδ T-cell receptor.


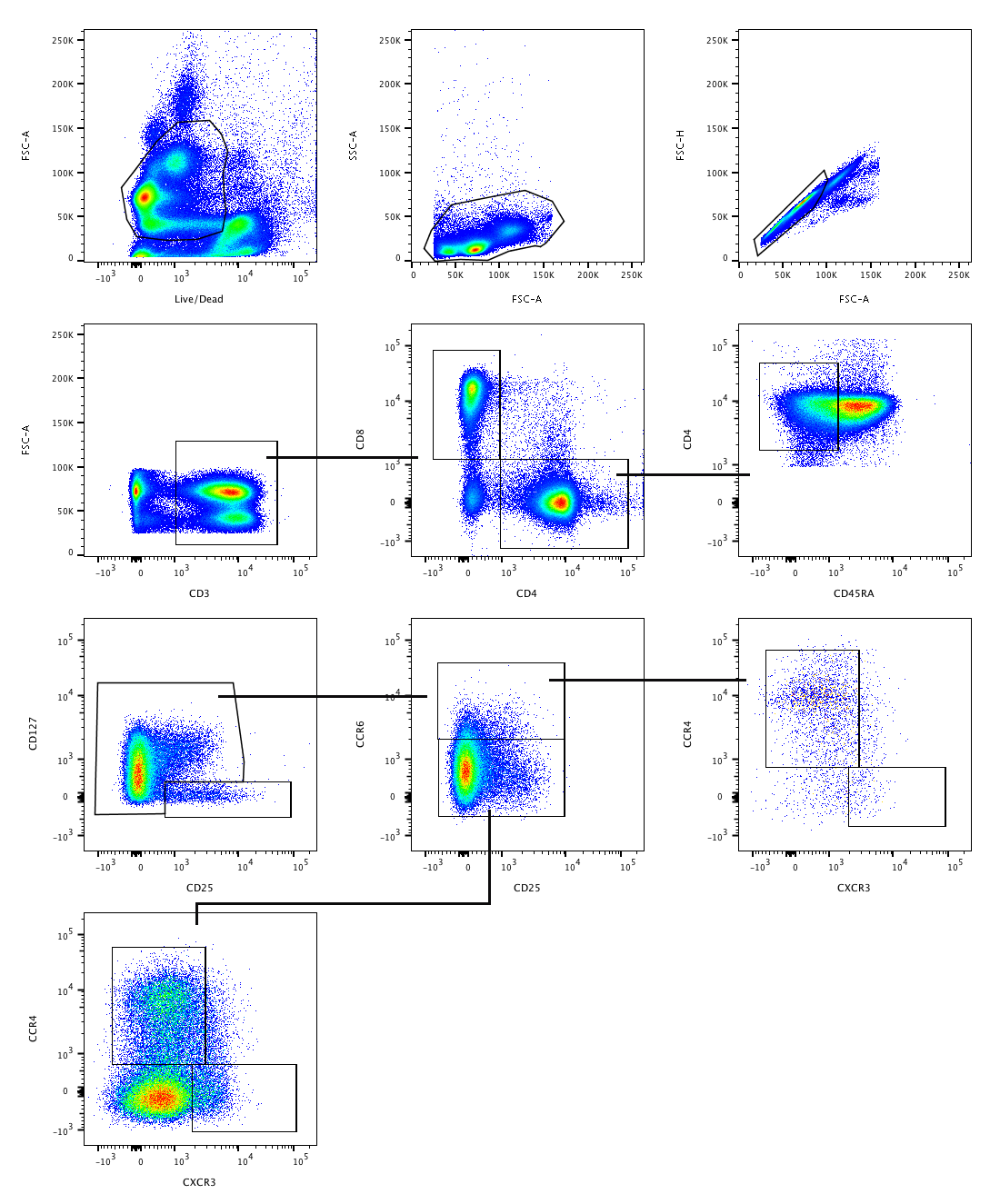


**Supplementary Figure 11: Gating strategy to determine Th-cell subsets in Cohort B samples.**


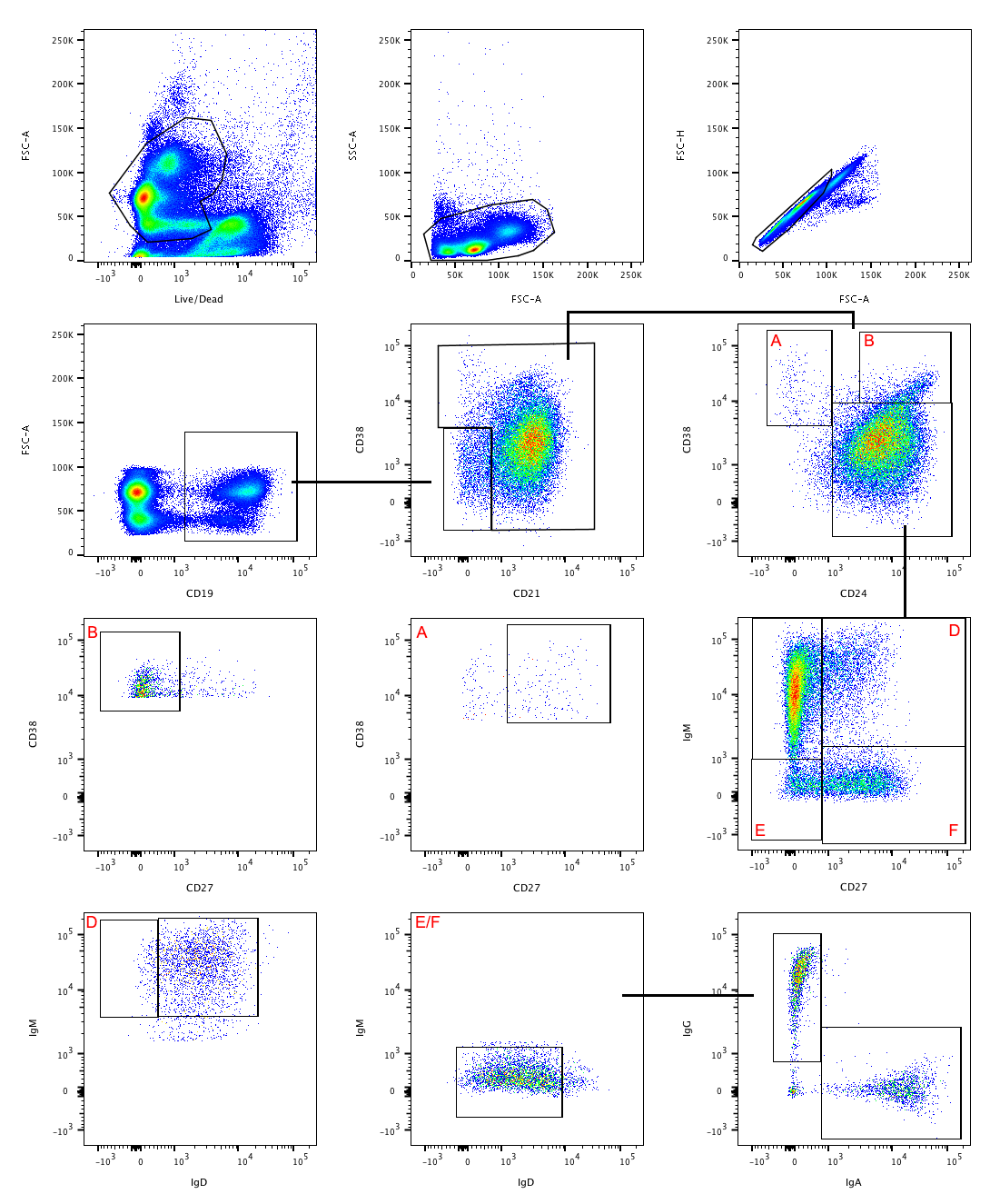


**Supplementary Figure 12: Gating strategy to determine B-cell subsets in Cohort B samples.**

**Supplementary Table 1: List of antibodies used in this study.** Details of clones and suppliers: Laksono BM, *et al*. In vitro measles virus infection of human lymphocyte subsets demonstrates high susceptibility and permissiveness of both naive and memory B-cells. J Virol 92, 8 (2018).

| **Fluorochrome** | **Cohort A** | **Cohort B** | | |
| --- | --- | --- | --- | --- |
|  |  | **Staining Set 1** | **Staining Set 2** | **Staining Set 3** |
| BV421 | CD27 (1:25; BD Biosciences, cat. no. 562513) | CD27 (1:25; BD Biosciences, cat. no. 562513) | CD27 (1:10; BD Biosciences, cat. no. 562513) | CD25 (1:20; BioLegend, cat. no. 302630) |
| Aqua | LIVE/DEAD (1:50; Life Technologies, cat. no. L34967) |  |  |  |
| BV510 |  | IgM (1:20; BioLegend, cat. no. 314522) | CD4 (1:30; BioLegend, cat. no. 317444) | CD4 (1:30; BioLegend, cat. no. 317444) |
| BV605 | CD45RO (1:250; BioLegend, cat. no. 304238) | CD38 (1:30; BioLegend, cat. no. 303532) | CD45RA (1:250; BioLegend, cat. no. 304134) | CD45RA (1:10; BioLegend, cat. no. 304134) |
| BV711 | Isotype (1:50; BioLegend, cat. no. 400354) |  |  | CXCR3 (1:10; BD Biosciences, cat. no. 563156) |
| FITC | MV nucleoprotein (1:250; Merck Millipore, cat. no. MAB8906F) | Viability dye 520 (1:1,000; BD Biosciences, cat. no. 564407) | Viability dye 520 (1:1,000; BD Biosciences, cat. no. 564407) | Viability dye 520 (1:1,000; BD Biosciences, cat. no. 564407) |
| PerCP-Cy5.5 | IgD (1:10; BioLegend, cat. no. 348208) | IgD (1:10; BioLegend, cat. no. 348208) | CD28 (1:35; BioLegend, cat. no. 302922) | CCR6 (1:20; BioLegend, cat. no. 353406) |
| PE | CD3 (1:10; BD Biosciences, cat. no. 345765) | IgA (1:10, Miltenyi Biotec, cat. no. 130-093-128) | CD3 (1:10; BD Biosciences, cat. no. 345765) | CD3 (1:40; BD Biosciences, cat. no. 345765) |
| PE-CF594 | CCR7 (1:20; BD Biosciences, cat. no. 562381) | IgG (1:50; BD Biosciences, cat. no. 562538) | CCR7(1:20; BD Biosciences, cat. no. 562381) |  |
| PE-Cy7 | CD19 (1:10; Beckman Coulter, cat. no. IM3628) | CD19 (1:10; Beckman Coulter, cat. no. IM3628) | TCRγδ (1:50; BD Biosciences, cat. no. 655410) | CCR4 (1:10; BioLegend, cat. no. 359410) |
| APC | CD4 (1:10; BD Biosciences, cat. no. 555349) | CD21 (1:10; BD Biosciences, cat. no. 559867) | CXCR5 (1:5; R&D Systems, cat. no. FAB190A-100) | CD127 (1:20; BioLegend, cat. no. 351316) |
| APC-H7 | CD8 (1:10; BD Biosciences, cat. no. 641400) | CD24 (1:10; Beckman Coulter, cat. no. PN B10738) | CD8 (1:20; BD Biosciences, cat. no. 641400) | CD8 (1:10; BD Biosciences, cat. no. 641400) |
