# Supplementary material for: Studies into the mechanism of measles-associated immune suppression during a measles outbreak in the Netherlands
Source: Nat Commun. 2018 Nov 23;9:4944. doi: 10.1038/s41467-018-07515-0 (PMC6251901; doi:10.1038/s41467-018-07515-0)
Supplement: Supplementary file 2 — Description of Additional Supplementary Files [file 41467_2018_7515_MOESM2_ESM.docx]

**Description of Additional Supplementary Files**

**File Name**: Supplementary Data 1

**Description**: Acute measles patient data.

**File Name**: Supplementary Data 2

**Description**: Cohort B details.

**File Name**: Supplementary Data 3

**Description**: Clinical protocol of “Studies into the mechanism of measles-associated immune suppression during an outbreak of measles in The Netherlands” (NL45323.078.13/2).
