# Supplementary material for: Studies into the mechanism of measles-associated immune suppression during a measles outbreak in the Netherlands
Source: Nat Commun. 2018 Nov 23;9:4944. doi: 10.1038/s41467-018-07515-0 (PMC6251901; doi:10.1038/s41467-018-07515-0)
Supplement: Supplementary file 5 — Supplementary Data 3 [file 41467_2018_7515_MOESM5_ESM.pdf]

Studies into the mechanism of measles-associated immune suppression during an outbreak of measles in The Netherlands

(Version 2, 9 July 2013)

See Dutch CCMO register:

https://www.toetsingonline.nl/to/ccmo_search.nsf/Searchform?OpenForm

Search for dossier nummer "NL45323.078.13"

PROTOCOL TITLE '**Studies into the mechanism of measles-associated immune suppression during an outbreak of measles in The Netherlands**'

**Protocol ID**	**NL45323.078.13**
**Short title**	**How does measles cause immune suppression?**
**EudraCT number**	***Not applicable***
**Version**	**2**
**Date**	**09-07-2013**
**Coordinating investigator/project leader**	***Not applicable***
**Principal investigator(s) (in Dutch: hoofdonderzoeker/ uitvoerder)**	***Dr. Rik L. de Swart***<br>***Erasmus MC, Dept. Viroscience, Room Ee1722A***<br>***'s-Gravendijkwal 230, 3015 CE Rotterdam***<br>***Tel+31 10 7044280; Fax +31 10 7044760***<br>**E-mail r.deswart@erasmusmc.nl**
**Sponsor (in Dutch: verrichter/opdrachtgever)**	***Prof. A.D.M.E. Osterhaus***
**Subsidising party**	***Erasmus MC, Dept. Viroscience***<br>***'s-Gravendijkwal 230, 3015 CE Rotterdam***<br>***Tel +31 10 7044066***<br>***E-mail a.osterhaus@erasmusmc.nl***
**Independent expert**	***Dr. Arthur M. Bohnen***<br>***Erasmus MC, Dept. General Practice***<br>***'s-Gravendijkwal 230, 3015 CE Rotterdam***<br>**Tel +31 10 7032115 / 7043747**<br>**E-mail a.bohnen@erasmusmc.nl**
**Laboratory sites**	***Erasmus MC, Dept. Viroscience***<br>***'s-Gravendijkwal 230, 3015 CE Rotterdam***
**Pharmacy**	***Not applicable***

PROTOCOL SIGNATURE SHEET

Name	Signature	Date
**Sponsor or legal representative:**<br>*Not applicable*<br><br>*<For non-commercial research,>*<br>**Head of Department:**<br>*Prof. A.D.M.E. Osterhaus*		
**[Coordinating Investigator/Project leader/Principal Investigator]:**<br>*Dr. R.L. de Swart*		

TABLE OF CONTENTS

OBJECTIVES 12
STUDY DESIGN 13
1. STUDY POPULATION 15
1.1 Population (base) 15
The study population consists of children from families in the orthodox Protestant ‘bible belt’ community. Families willing to participate in this study will be identified with the help of infectious disease specialists from the Municipal Health offices in the area. We intend to approach orthodox protestant schools with low vaccination coverage to approach these families. 15
1.2 Inclusion criteria 15
1.3 Exclusion criteria 15
1.4 Sample size calculation 15
2. TREATMENT OF SUBJECTS 17
2.1 Investigational product/treatment 17
2.2 Use of co-intervention (if applicable) 17
2.3 Escape medication (if applicable) 17
3. INVESTIGATIONAL PRODUCT 18
3.1 Name and description of investigational product(s) 18
3.2 Summary of findings from non-clinical studies 18
3.3 Summary of findings from clinical studies 18
3.4 Summary of known and potential risks and benefits 18
3.5 Description and justification of route of administration and dosage 18
3.6 Dosages, dosage modifications and method of administration 18
3.7 Preparation and labelling of Investigational Medicinal Product 18
3.8 Drug accountability 18
4. NON-INVESTIGATIONAL PRODUCT 19
4.1 Name and description of non-investigational product(s) 19
4.2 Summary of findings from non-clinical studies 19
4.3 Summary of findings from clinical studies 19
4.4 Summary of known and potential risks and benefits 19
4.5 Description and justification of route of administration and dosage 19
4.6 Dosages, dosage modifications and method of administration 19
4.7 Preparation and labelling of Non Investigational Medicinal Product 19
4.8 Drug accountability 19
5. METHODS 20
5.1 Study parameters/endpoints 20
5.1.1 Main study parameter/endpoint 20
5.1.2 Secondary study parameters/endpoints (if applicable) 20
5.1.3 Other study parameters (if applicable) 20
5.2 Randomisation, blinding and treatment allocation 20
5.3 Study procedures 21

5.4 Withdrawal of individual subjects 21
5.4.1 Specific criteria for withdrawal (if applicable) 21
5.5 Replacement of individual subjects after withdrawal 21
5.6 Follow-up of subjects withdrawn from treatment 21
5.7 Premature termination of the study 21
SAFETY REPORTING 22
5.8 Section 10 WMO event 22
5.9 AEs, SAEs and SUSARs 22
5.9.1 Adverse events (AEs) 22
5.9.2 Serious adverse events (SAEs) 22
5.9.3 Suspected unexpected serious adverse reactions (SUSARs) 23
5.10 Annual safety report 23
5.11 Follow-up of adverse events 23
5.12 [Data Safety Monitoring Board (DSMB) / Safety Committee] 23
6. STATISTICAL ANALYSIS 24
6.1 Primary study parameter(s) 24
6.2 Secondary study parameter(s) 25
6.3 Other study parameters 25
6.4 Interim analysis (if applicable) 25
7. ETHICAL CONSIDERATIONS 26
7.1 Regulation statement 26
7.2 Recruitment and consent 26
7.3 Objection by minors or incapacitated subjects (if applicable) 27
7.4 Benefits and risks assessment, group relatedness 27
7.5 Compensation for injury 28
7.6 Incentives (if applicable) 28
8. ADMINISTRATIVE ASPECTS, MONITORING AND PUBLICATION 29
8.1 Handling and storage of data and documents 29
8.2 Monitoring and Quality Assurance 29
8.3 Amendments 29
8.4 Annual progress report 30
8.5 End of study report 30
8.6 Public disclosure and publication policy 30
9. STRUCTURED RISK ANALYSIS 31
9.1 Potential issues of concern 31
9.2 Synthesis 31
10. REFERENCES 32

LIST OF ABBREVIATIONS AND RELEVANT DEFINITIONS

ABR	ABR form, General Assessment and Registration form, is the application form that is required for submission to the accredited Ethics Committee (In Dutch, ABR = Algemene Beoordeling en Registratie)
AE	Adverse Event
AR	Adverse Reaction
CA	Competent Authority
CCMO	Central Committee on Research Involving Human Subjects; in Dutch: Centrale Commissie Mensgebonden Onderzoek
CV	Curriculum Vitae
DSMB	Data Safety Monitoring Board
EU	European Union
EudraCT	European drug regulatory affairs Clinical Trials
IC	Informed Consent
METC	Medical research ethics committee (MREC); in Dutch: medisch ethische toetsing commissie (METC)
MV	Measles virus
PBMC	Peripheral blood mononuclear cells
(S)AE	(Serious) Adverse Event
Sponsor	The sponsor is the party that commissions the organisation or performance of the research, for example a pharmaceutical company, academic hospital, scientific organisation or investigator. A party that provides funding for a study but does not commission it is not regarded as the sponsor, but referred to as a subsidising party.
SUSAR	Suspected Unexpected Serious Adverse Reaction
Wbp	Personal Data Protection Act (in Dutch: Wet Bescherming Persoonsgevens)
WMO	Medical Research Involving Human Subjects Act (in Dutch: Wet Medisch-wetenschappelijk Onderzoek met Mensen

SUMMARY

Rationale: Measles remains an important vaccine-preventable cause of morbidity and mortality. Hallmark of the disease is a transient but severe immune suppression, which results in increased susceptibility to opportunistic infections. In the Netherlands, children are usually vaccinated against measles at the ages of 14 months and 9 years. However, a group of socio-geographically clustered orthodox Protestants refuses vaccination on religious grounds. As a result, outbreaks of infectious diseases are repeatedly observed in this community. The last measles outbreak occurred in 1999/2000, and a new outbreak has started in June 2013.

Objective: Using an animal model for measles, we have recently developed a hypothesis to explain the mechanism of measles immune suppression. We hypothesize that MV causes massive infection and subsequent depletion of memory lymphocytes, and thus largely wipes out immunological memory. The objective of the current study is to validate this hypothesis in naturally infected measles patients: we will verify if MV predominantly infects memory lymphocytes, and if this results in depletion of pre-existing memory lymphocytes.

Study design: Observational cohort study

Study population: Unvaccinated children in orthodox protestant families, 4-17 years of age. This is the only age group eligible for this study, as older children or adults will be immune to measles.

Intervention (if applicable): Not applicable

Main study parameters/endpoints: The study consists of two cohorts. In cohort A clinical specimens (a blood sample and swabs of the upper respiratory tract) will be collected from children with an acute MV infection, shortly before, at or after onset of rash. The main study parameter in this group will be the characterization of MV-infected lymphocytes in peripheral blood, in order to confirm that the virus predominantly infects memory T- and B-lymphocytes. In cohort B blood samples will be collected from children that have not yet been exposed to MV. A second blood sample will be collected two to three weeks after recovery from measles. The main study parameter in this group will be the quantification of pre-existing memory lymphocyte populations in peripheral blood, to test the hypothesis that these cell populations are decimated during measles.

Nature and extent of the burden and risks associated with participation, benefit and group relatedness: The children will be asked to provide one (cohort A) or two (cohort B) blood samples. They will be visited in their homes, and will not undergo physical examinations or other tests. The study does not result in benefits to the participating children. However, the risks are minimal and the study can only be performed in this age group and this community due to the fact that the vast majority of adults in the Netherlands are immune to measles.

INTRODUCTION AND RATIONALE

Introduction

Measles remains an important vaccine-preventable cause of morbidity and mortality. The causative agent, measles virus (MV), is one of the most contagious human viruses known, and is transmitted via aerosols or direct contact with contaminated respiratory secretions. The virus causes a systemic disease with clinical signs that appear approximately two weeks after primary infection and include fever, rash, cough, coryza and conjunctivitis.[1] While significant progress has been made in global control programs, 139,000 deaths were still attributed to measles in 2010.[2] In the Netherlands, national measles epidemics occur due to vaccination refusal in a group of socio-geographically clustered orthodox Protestants. In 1999 the last measles epidemic in the Netherlands led to 3292 measles patients, of which 16% developed complications and 3 died.[3] Over the last decade, identification of new cellular receptors and studies in animal models with recombinant MV strains have challenged the historic concepts of measles pathogenesis.

Pathogen

MV belongs to the family *Paramyxoviridae*, genus *Morbillivirus*. Virus particles are pleiomorphic and average in size from 100 – 300 nm.[1] The viral genome consists of a single-stranded RNA molecule of negative polarity, typically 15,894 nucleotides in length, consisting of 6 genes that encode 8 proteins. The genome is contained in a helical nucleocapsid, which is surrounded by a lipid bilayer.[4] The envelope is derived from the membrane of the infected cell during budding.[5]

Clinical features and epidemiology

After infection via aerosol inhalation, MV replicates in the lymphoid tissues of the respiratory tract. After a relatively long incubation phase, patients develop fever and upper respiratory tract symptoms. The hallmark of measles is a maculopapular rash that appears around 14 days post infection,[6] starting behind the ears and eventually covering the entire body. Within a few days symptoms usually start to subside, and in absence of further complications patients recover rapidly. Measles is associated with immune suppression, leading to an increased susceptibility to opportunistic infections. This explains why a significant percentage of measles patients develop complications, resulting in a plethora of clinical symptoms.[7-10] For instance, bacterial pneumonia is a common complication and a major cause of measles-associated deaths.[11] Mortality rates may reach 25% in refugee camps or overcrowded populations, 5 – 10% in developing countries, but is usually less than 0.1% in industrialized countries.[12,13]

Animal models and recombinant MV strains

Small laboratory animals poorly reproduce the normal course of MV infection in humans. However, monkeys are highly sensitive to infection with MV and develop clinical signs and pathologic lesions similar to those described in humans.[14-18] Moreover, natural infections following contact of non-human primates with MV-infected humans have frequently been reported.[18,19] The combination of a highly susceptible non-human primate animal model and virulent rMV strains expressing fluorescent reporter proteins[20] has allowed detailed studies of the full spectrum of measles pathogenesis.

Pathogenesis studies in monkeys

Using recombinant MV strains expressing EGFP, we have previously demonstrated that MV predominantly infects $CD150^+$ lymphocytes and dendritic cells *in vivo*.[21] Subsequently, we have demonstrated that MV enters the host by infection of myeloid cells in the respiratory tract. Upon aerosol infection of macaques with a high dose of MV, the earliest MV-infected cells were alveolar macrophages and dendritic cells in the alveolar spaces of the lungs.[22] After initial replication in the lungs and lymphoid organs MV also spreads to other non-lymphoid tissues, including the gingiva, tongue, buccal mucosa, trachea, nose and skin, with infection usually starting in lymphoid aggregates within these tissues. MV dissemination mainly takes place in the form of MV-infected cells, rather than as cell-free virus. Transmission to the next host is mediated by MV produced by infected epithelial cells in the nose or MV-infected lymphocytes from the tonsils and adenoids that gain access to the upper respiratory tract due to MV-mediated epithelial damage.[22-24] During coughing both cell-free and cell-associated MV are expelled into the air, which can be inhaled by a next susceptible host.

Measles-associated immune suppression

Measles infection leads to immune activation[25] and robust MV-specific humoral and cellular immune responses, resulting in life-long protection.[11] However, the disease is also associated with a transient but profound immune suppression, an apparent contradiction usually referred to as the measles paradox. Measles immune suppression may last for several weeks to months after the acute stage of the disease, and thus extends beyond the lymphopenia usually observed during acute measles.[1] The clinical importance of measles immune suppression is illustrated by the observation that measles mortality is typically caused by secondary infections in the respiratory or digestive tracts.[7,10,26] However, the mechanisms by which MV causes immune suppression are not completely understood. Multiple *in vivo* correlates of immune suppression have been described, including

disappearance of Mantoux responses,[27,28] lymphopenia[29,30] and impaired responses to vaccination.[31,32] Decreased lymphoproliferative responses,[33-35] altered cytokine profiles[36,37] and impairment of antigen-presenting cell function[38-40] have been described *in vitro*. The relevance of these observations to immune suppression and enhanced susceptibility to opportunistic infections remains unclear.

It has been hypothesized that preferential infection of memory lymphocytes also plays a role in the measles-associated immune suppression.[21,41] Our studies have shown that measles immune suppression can, at least in part, be explained by massive infection and subsequent immune-mediated clearance of $CD150^+$ memory T-lymphocytes and follicular B-lymphocytes.[42] MV preferentially infected $CD45RA^-$ central memory and effector memory, which during secondary immune responses are the primary source of T-lymphocyte expansion or generation of effector T-lymphocytes, respectively.[43] Infection and subsequent immune-mediated depletion of memory T-lymphocyte subsets fits with the first description of measles-induced immune suppression, namely the disappearance of Mantoux responses in measles patients.[27] The novelty of our immune suppression model lies in an immune-mediated lymphodepletion being masked by the massive expansion of MV-specific and bystander lymphocytes.

Measles in the Dutch orthodox protestant community

Despite high vaccination coverage in the general population, outbreaks of infectious diseases continue to occur in The Netherlands. These outbreaks are largely confined to the orthodox Protestant minority. This is a religious minority of approximately 250,000 people, who have religious objections to vaccination and form a socio-geographically clustered community.[44] Measles outbreaks have been observed in this community in 1976, 1983, 1987-1988, 1992-1994 and 1999-2000.[45] Since the year 2000 no measles outbreaks have been reported in this community, and it must be assumed that all children that were born since then and have not been vaccinated are fully susceptible to MV infection. Therefore, valid studies in naive and non-measles exposed persons are restricted to children born after the 2000 measles outbreaks.

Although other small and large measles outbreaks have been observed in the general population of The Netherlands since 2000,[46] these have not spread to the so-called bible belt. However, early 2013 a number of measles cases have been diagnosed in children with an orthodox protestant background, who also attended a school with low vaccination coverage. Based on historical observations, this suggested that a large measles outbreak may be imminent in this community. Indeed, since the beginning of June 2013 a large

number of measles cases has been observed in the community, and a first meeting of a measles ‘outbreak management team’ (OMT) is organized on Monday June 17.

Such an outbreak constitutes a unique opportunity to test our hypothesis on the mechanism of measles immune suppression, as it allows identification of children that are highly likely to develop measles in a forthcoming measles outbreak. As such, this allows collection of pre- and post-exposure blood samples, providing a unique opportunity to measure the frequency of specific memory lymphocyte populations before and after measles. These studies will contribute to our understanding of the pathogenesis of measles and the associated immunosuppression.

OBJECTIVES

Primary Objective (Cohort A and B): To test the hypothesis that MV preferentially infects memory lymphocytes, resulting in immune-mediated depletion of these cells leading to immunological amnesia. In cohort A this will be investigated by determining the phenotype and frequency of MV infected white blood cells in the blood of acute measles patients, using immunofluorescence staining. In cohort B we will determine the size of pre-existing memory lymphocyte subpopulations pre- and post-measles.

Secondary Objective(s): As a secondary objective, we will try to validate and translate observations from our animal model studies to natural measles virus infections in children.

To this end, we will perform the following studies:

Cohort A:

- Measure MV-specific antibody concentrations in the plasma of acute measles patients;
- Measure diagnostic haematological parameters, including numbers (and differentiation) of white blood cells, red blood cells, platelets;
- Measure cytokine, chemokine and acute phase protein concentrations in the plasma of acute measles patients, as correlates of the ongoing MV-specific immune response;
- Assess the shape and size of MV particles directly in throat- and nose swabs and in PBMC, using electron microscopy;
- Determine viral loads in throat- and nose swabs and PBMC of acute MV patients by virus isolation and RT-PCR;
- Determine the difference between cell-free and cell-associated MV in throat- and nose-swabs;

Cohort B:

- Identify tetramers that can be used to measure specific memory subpopulations in PBMC pre-exposure to measles;
- Measure and characterize MV-specific cellular immune responses in PBMC;
- Measure MV-specific antibody concentrations in plasma samples;
- Measure diagnostic haematological parameters, including numbers (and differentiation) of white blood cells, red blood cells, platelets;
- Measure expansion and/or depletion of lymphocyte subpopulations in relation to MV infection, by T-cell receptor rearrangement studies;

STUDY DESIGN

This is an observational study, consisting of two cohorts.

Cohort A consists of unvaccinated children aged 4-17 years. These children will be recruited in orthodox Protestant families in which at least one child has been diagnosed with measles. Families willing to participate in this study will be identified with the help of Municipal Health infectious disease specialists in the orthodox Protestant community region and/or based on registered cases in the RIVM-Osiris database. Based on age, vaccination history and disease history, it will be possible to identify family members of the patient that are likely in the incubation phase of the disease if they are within the same age range defined for this cohort. We will attempt to collect clinical specimens in the prodromal phase before the onset of rash: this is the period that percentages MV-infected cells in peripheral blood reach the highest levels. After obtaining informed consent, the following clinical specimens will be collected: throat swab, nose swab and heparinised blood sample (single tube, maximum volume 9 ml).

The respiratory swabs will be used for virus detection, which will give an indication of the stage of MV infection the children are in. Peripheral blood mononuclear cells (PBMC) will be isolated from the blood sample and will be used for MV isolation and for staining of MV-infected cells by flow cytometry. By co-staining with different monoclonal antibodies, we will quantify the levels of MV infection in different T- and B-lymphocyte populations. Development of disease symptoms will be recorded by the parents, so that the infection percentages can be plotted on a time line relative to the onset of rash.[47]

Cohort B consists of unvaccinated children aged 6-13 years. These children will be recruited by contacting orthodox protestant schools with low vaccination coverage. Via the schools (groups 3-8) we will approach families that have not yet been exposed to MV, but live in an area that is expected to become part of the measles outbreak. One tube (maximum volume 9 ml) of heparinised blood will be collected; PBMC will be isolated and frozen. The parents will be contacted every two weeks by email or telephone, to monitor whether the children have contracted measles. Two to three weeks after the end of the measles outbreak in the school, a second 9 ml heparinised blood sample will be collected from the same children. This second blood sample will also be collected if the child did not experience measles during this outbreak. In this case the child will serve as an internal control: according to our hypothesis the frequency of pre-existing memory lymphocytes should remain stable in children that do not develop measles. However, we estimate the chances of unprotected children escaping from measles virus infection at a school with low vaccination coverage as minimal. The main

study parameter in this group will be the quantification of pre-existing memory lymphocyte populations in peripheral blood by tetramer staining, to test the hypothesis that these populations are decimated during measles.

1. STUDY POPULATION

1.1 Population (base)

The study population consists of children from families in the orthodox Protestant 'bible belt' community. Families willing to participate in this study will be identified with the help of infectious disease specialists from the Municipal Health offices in the area. We intend to approach orthodox protestant schools with low vaccination coverage to approach these families.

1.2 Inclusion criteria

In order to be eligible to participate in this study, a subject must meet all of the following criteria:

- Aged 4 – 17 years old
- Unvaccinated against measles
- No known history of natural measles

1.3 Exclusion criteria

A potential subject who meets any of the following criteria will be excluded from participation in this study:

- Chronic disease
- Immune suppression (due to medication or underlying disease)

1.4 Sample size calculation

Cohort A:

To obtain significant percentages and proof of lymphocyte memory subset infection by MV, we anticipate that we need PBMC samples of at least 25 children who are in the incubation phase of measles and have detectable levels of infected cells in their PBMC. Using multi-color flow cytometry we will identify the phenotype of the infected cells. However, there are uncertain variables that need to be taken into account.

The clinical specimens that are required to meet our study objective need to be collected during the incubation phase of the disease, when neither clinical nor laboratory diagnosis of measles is possible at the bedside. Therefore, it is a possibility that children that prove not to be MV-infected are included in this cohort. Furthermore, MV infection causes lymphopenia. As a result, isolation of PBMC from blood may in some cases not result in

sufficient numbers of cells for the flow cytometric staining to characterize the MV-infected subsets.

It is difficult to estimate how many samples will be lost due to one of these two causes. However, since both the laboratory diagnosis and the quality and quantity of PBMC are available within two days after collection of clinical specimens, we will be able to monitor the build-up of data in this cohort in real time. As soon as we have the required 25 successful PBMC stains of laboratory-confirmed measles cases, we will stop recruiting children to this cohort. In total, we will not include more than 100 children in this cohort.

Cohort B:

To obtain significant data in cohort B, we require paired pre- and post-measles PBMC of at least 50 children. This will allow statistical comparison of the precursor frequencies of circulating memory lymphocyte populations before and after measles.

Here, we need to take other uncertainties into account. Most importantly, it is a possibility that children that consent to collection of a pre-measles blood sample may decide to withdraw their consent for collection of a second sample. Historically, it has been observed that this occurs in approximately 50% of the children. An additional theoretical risk is that the school of the children does not become involved in the measles outbreak. However, observations from previous measles outbreaks in the bible belt suggest that this risk is low, especially since we intend to recruit children at schools with very low vaccination coverage. Finally, the detection of memory lymphocyte populations by flow cytometry will determine on the availability of suitable tetramers that can be used to stain these populations by flow cytometry. Tetramers are restricted to specific HLA-haplotypes, and thus technical limitations may result in the inability to detect memory lymphocytes in a specific patient.

As a result, we estimate that it will be necessary to collect a first blood sample of 100 children.

2. TREATMENT OF SUBJECTS

Not applicable

2.1 Investigational product/treatment

2.2 Use of co-intervention (if applicable)

2.3 Escape medication (if applicable)

3. INVESTIGATIONAL PRODUCT

Not applicable

3.1 Name and description of investigational product(s)

3.2 Summary of findings from non-clinical studies

3.3 Summary of findings from clinical studies

3.4 Summary of known and potential risks and benefits

3.5 Description and justification of route of administration and dosage

3.6 Dosages, dosage modifications and method of administration

3.7 Preparation and labelling of Investigational Medicinal Product

3.8 Drug accountability

4. NON-INVESTIGATIONAL PRODUCT

Not applicable

4.1 Name and description of non-investigational product(s)

4.2 Summary of findings from non-clinical studies

4.3 Summary of findings from clinical studies

4.4 Summary of known and potential risks and benefits

4.5 Description and justification of route of administration and dosage

4.6 Dosages, dosage modifications and method of administration

4.7 Preparation and labelling of Non Investigational Medicinal Product

4.8 Drug accountability

5. METHODS

5.1 Study parameters/endpoints

5.1.1 Main study parameter/endpoint

Cohort A: the main study parameter of this cohort is the phenotypic characterization of MV-infected white blood cells by multicolour flow cytometry.

Cohort B: the main study parameters in this cohort are the identification of pre-existing memory lymphocyte populations in peripheral blood before MV infection (by tetramer staining in combination with multicolour flow cytometry) and the change in the frequency of these populations following MV infection.

5.1.2 Secondary study parameters/endpoints (if applicable)

Cohort A: secondary study parameters in cohort A are the identification and characterization of cell-free and cell-associated virus, both in terms of quantity (what virus concentrations are present in the upper respiratory tract in relation to transmissibility of MV) and quality (what does transmissible MV look like under the electron microscope?). In addition, we will investigate what cytokines, chemokines and acute phase proteins can be measured in the plasma during the early stage of acute MV infection, in relation to the level of lymphopenia (reduced lymphocyte numbers in peripheral blood).

Cohort B: secondary parameters in cohort B are the measurement and characterization of MV-specific humoral and cellular immune responses. Massive expansion of MV-specific (and bystander) B- and T-lymphocyte populations is an essential characteristic of our immune suppression model, so we will investigate whether the quantitative and qualitative composition of the circulating lymphocyte pool of children after measles has indeed changed when compared to the pre-infection sample, while the composition of the paired samples of the control children remains essentially unaltered.

5.1.3 Other study parameters (if applicable)

Not applicable

5.2 Randomisation, blinding and treatment allocation

Not applicable.

5.3 Study procedures

Cohort A: children will undergo venepuncture in the presence of a parent during the acute phase of measles, to collect one tube of approximately 9 ml heparinised blood. In addition, a throat swab and a nose swab will be collected in virus transport medium. In Cohort B children will undergo venepuncture at two time points, but no other clinical specimens will be collected. These children are in normal health at the moment of sample collection. The blood sampling can be considered as a negligible risk procedure. The procedure will be performed by a skilled person. In addition, all children will be offered EMLA as a local anaesthetic to reduce pain during the venepuncture. Obtaining a throat- and nose swab can be inconvenient for children and the nose swab may cause a minor nose bleed.

5.4 Withdrawal of individual subjects

Subjects can leave the study at any time for any reason if they wish to do so without any consequences. The investigator can decide to withdraw a subject from the study for urgent medical reasons. Minors cannot be forced to undergo interventions against his or her will. This study will be conducted according to the Code of Conduct applicable to minors as written by the Paediatric Association of the Netherlands (Nederlandse Vereniging voor Kindergeneeskunde). The parents will be informed about the possibility to withdraw their children at any moment in the study without consequences for the medical care in an information letter they will receive upon inclusion.

5.4.1 Specific criteria for withdrawal (if applicable)

Not applicable.

5.5 Replacement of individual subjects after withdrawal

If parents have consented but children refuse to cooperate before any samples have been collected, the subject will be replaced. If samples have already been collected the subject will remain included.

5.6 Follow-up of subjects withdrawn from treatment

Not applicable.

5.7 Premature termination of the study

Not applicable.

SAFETY REPORTING

5.8 Section 10 WMO event

In accordance to section 10, subsection 1, of the WMO, the investigator will inform the subjects and the reviewing accredited METC if anything occurs, on the basis of which it appears that the disadvantages of participation may be significantly greater than was foreseen in the research proposal. The study will be suspended pending further review by the accredited METC, except insofar as suspension would jeopardise the subjects' health. The investigator will take care that all subjects are kept informed.

5.9 AEs, SAEs and SUSARs

5.9.1 Adverse events (AEs)

Adverse events are defined as any undesirable experience occurring to a subject during the study, whether or not considered related to [the investigational product / the experimental interventiont]. All adverse events reported spontaneously by the subject or observed by the investigator or his staff will be recorded.

5.9.2 Serious adverse events (SAEs)

A serious adverse event is any untoward medical occurrence or effect that at any dose:

- results in death;
- is life threatening (at the time of the event);
- requires hospitalisation or prolongation of existing inpatients' hospitalisation;
- results in persistent or significant disability or incapacity;
- is a congenital anomaly or birth defect;
- Any other important medical event that may not result in death, be life threatening, or require hospitalization, may be considered a serious adverse experience when, based upon appropriate medical judgement, the event may jeopardize the subject or may require an intervention to prevent one of the outcomes listed above.

The principal investigator will report the SAEs through the web portal *ToetsingOnline* to the accredited METC that approved the protocol, within 15 days after the principal investigator has first knowledge of the serious adverse reactions.

SAEs that result in death or are life threatening should be reported expedited. The expedited reporting will occur not later than 7 days after the responsible investigator

has first knowledge of the adverse reaction. This is for a preliminary report with another 8 days for completion of the report.

5.9.3 Suspected unexpected serious adverse reactions (SUSARs)

Not applicable.

5.10 Annual safety report

Not applicable.

5.11 Follow-up of adverse events

All AEs will be followed until they have abated, or until a stable situation has been reached. Depending on the event, follow up may require additional tests or medical procedures as indicated, and/or referral to the general physician or a medical specialist. SAEs need to be reported till end of study within the Netherlands, as defined in the protocol.

5.12 [Data Safety Monitoring Board (DSMB) / Safety Committee]

Not applicable

6. STATISTICAL ANALYSIS

In cohort A the outcomes will be presented as qualitative data: the percentage MV-infected cells within different white blood cell populations and subpopulations will be determined by flow cytometry. These data will be linked to the time point of sample collection, expressed as the number of days before or after onset of rash. To test our hypothesis that MV predominantly infects memory lymphocytes, the percentages MV-infected cells within the memory B-lymphocyte, memory CD4+ T-lymphocyte and memory CD8+ T-lymphocyte subpopulations will be compared with those in the naïve B-lymphocyte, CD4+ T-lymphocyte and CD8+ T-lymphocyte subpopulations, respectively. For this comparison we will use a paired t-test on log-transformed data. If the data are not normally distributed, a non-parametric test will be used for this comparison (Mann-Whitney U-test).

In cohort B the data will be presented as precursor frequencies of memory lymphocytes to infections unrelated to measles. The difference between these precursor frequencies before and after measles will be analysed by using a paired t-test on log-transformed data. If the data are not normally distributed, a non-parametric test will be used for this comparison (Mann-Whitney U-test). Patients or data will only be included if memory lymphocyte populations can be identified in the pre-exposure PBMC sample.

6.1 Primary study parameter(s)

Cohort A: the main study parameter of this cohort is the phenotypic characterization of MV-infected white blood cells by multicolour flow cytometry. Our hypothesis is that these will be predominantly memory lymphocytes. These data will be analysed as described above.

Cohort B: the main study parameters in this cohort are the identification of pre-existing memory lymphocyte populations in peripheral blood before MV infection (by tetramer staining in combination with multicolour flow cytometry) and the change in the frequency of these populations following MV infection. These data will be analysed as described above.

6.2 Secondary study parameter(s)

Cohort A: secondary study parameters in cohort A are the identification and characterization of cell-free and cell-associated virus, both in terms of quantity (what virus concentrations are present in the upper respiratory tract in relation to transmissibility of MV) and quality (what does transmissible MV look like under the electron microscope?). In addition, we will investigate what cytokines, chemokines and acute phase proteins can be measured in the plasma during the early stage of acute MV infection, in relation to the level of lymphopenia (reduced lymphocyte numbers in peripheral blood). These data will be described qualitatively, and do not require statistical analysis.

Cohort B: secondary parameters in cohort B are the measurement and characterization of MV-specific humoral and cellular immune responses. Massive expansion of MV-specific (and bystander) B- and T-lymphocyte populations is an essential characteristic of our immune suppression model; we will explore different methods to demonstrate that the qualitative composition of the circulating lymphocyte pool of children after measles is dramatically altered as compared to the pre-infection sample. If successful, these data will be analysed using a similar approach as above: by paired t-test on log-transformed data or by Mann-Whitney U-test if data are not normally distributed.

6.3 Other study parameters

Not applicable

6.4 Interim analysis (if applicable)

Not applicable

7. ETHICAL CONSIDERATIONS

7.1 Regulation statement

The study will be conducted according to the principles of the Declaration of Helsinki, as amended by the 59th WMA General Assembly, Seoul, Korea, October 2008, and in accordance with the Medical Research Involving Human Subjects Act (WMO) and other guidelines, regulations and Acts.

7.2 Recruitment and consent

Cohort A:

Measles is a notifiable disease and will be notified to the infectious disease specialists of the Municipal health Service. Municipal Health infectiologists residing in the Bible Belt area often have contacts in the orthodox Protestant community already. They will contact parents and ask them if they are interested to participate in the study. In addition, we will also inform orthodox Protestant General Practitioners of our research plans, by using our a page information sheet. If parents are potentially interested in allowing their children to participate in the study, we will provide them with our Letter of Consent. In this letter we ask them to decide within three days whether or not their children can participate, because the window of opportunity to collect samples during the incubation phase of the disease is relatively short. As soon as parents indicate they consent to the study we will visit them at home, and answer any additional questions they may have. After the parents (and potentially the children, if aged above 11) have signed the consent forms, we will collect the clinical specimens.

Cohort B:

In order to recruit subjects to cohort B, we intend to approach schools with low vaccination coverage (<30%) that have not yet seen measles cases in their school. We expect that measles will spread rapidly to all children in the school once the virus is introduced. Municipal Health infectiologists often have existing relationships with these schools, and can suggest which schools to approach. If the head of the school is convinced of the potential usefulness of the study, we will ask his permission to distribute the one page information sheet and the Informed Consent amongst children at the school. Parents who are willing to participate will fill in their contact information on the information sheet, which will be returned at the school and subsequently be collected by the research team. If the head of the school or the parents would consider this useful, we will try to organise an information session at the school, during which we can provide more background information about the virus, the disease and our clinical study. Parents who indicate they are willing to consent to the study will first be contacted by telephone, and subsequently be visited at home, and we will answer any additional questions they may have. After the parents (and potentially the children, if aged above 11) have signed the consent forms, we will collect the clinical specimens. Subsequently, the course of the

measles outbreak at the school will be monitored by the Municipal Health office. As soon as the outbreak at the school appears to be finished, we will start making arrangements for collection of the second blood sample. To this end, the children will again be visited at their home address. If the school and parents indicate they would prefer blood collection to take place at another location, we will investigate whether we can organize this either at the school or at a regional Municipal Health-associated location.

7.3 Objection by minors or incapacitated subjects (if applicable)

The informed consent letters will need to be signed by both parents. If the participating children are aged above 11, they will also need to sign themselves. However, even if parents have signed the informed consent letter, children who clearly indicate they do not want the clinical specimens to be collected will not be included in the study. We will strictly adhere to the ‘Code of conduct relating to expressions of objection by minors participating in medical research’ as established by the Netherlands Association for Paediatric Medicine (NVK) on 21 May 2001 and published in NVK Newsletter no. 3, June 2001.

7.4 Benefits and risks assessment, group relatedness

The study does not result in benefits to the participating children. However, the risks are minimal and the study can only be performed in this age group and this community due to the fact that the vast majority of individuals aged 14 and over in the Netherlands are immune to measles, either due to previous natural infection or vaccination. The bible belt population constitutes a unique community in the world, as previous outbreaks of infectious diseases, including measles, have been well documented. Based on these observations, we can be certain that all unvaccinated children in the orthodox Protestant community that live in this geographic region and go to schools with low vaccination grade will be infected with measles virus during this outbreak. In addition, the relatively large family size in orthodox Protestant families increase the chances of identifying families in which one child has already been diagnosed with measles, while his or her siblings are still in the incubation phase of measles virus infection. Together, the ongoing measles outbreak in the orthodox Protestant community provides us with a unique opportunity to perform these studies.

Although the results of this study are not of direct benefit to the participating children, it can be expected that more measles outbreaks may occur in this community in the future. Moreover, it will require years before potential plans to achieve global eradication of measles can be implemented. Therefore, a better understanding of the pathogenesis of

measles immune suppression will help in developing new intervention and treatment plans.

7.5 Compensation for injury

The sponsor/investigator has a liability insurance which is in accordance with article 7, subsection 6 of the WMO.

The sponsor (also) has an insurance which is in accordance with the legal requirements in the Netherlands (Article 7 WMO and the Measure regarding Compulsory Insurance for Clinical Research in Humans of 23th June 2003). This insurance provides cover for damage to research subjects through injury or death caused by the study.

1. € 450.000,-- (i.e. four hundred and fifty thousand Euro) for death or injury for each subject who participates in the Research;
2. € 3.500.000,-- (i.e. three million five hundred thousand Euro) for death or injury for all subjects who participate in the Research;
3. € 5.000.000,-- (i.e. five million Euro) for the total damage incurred by the organisation for all damage disclosed by scientific research for the Sponsor as ‘verrichter’ in the meaning of said Act in each year of insurance coverage.

The insurance applies to the damage that becomes apparent during the study or within 4 years after the end of the study.

7.6 Incentives (if applicable)

Parents of children will not receive an incentive to participate in the study. However, every child that fully participates in the study protocol will be provided with a git certificate with a value of 10 euro to thank them for their contribution.

8. ADMINISTRATIVE ASPECTS, MONITORING AND PUBLICATION

8.1 Handling and storage of data and documents

Data and materials will be labelled with an identification code. The key to that code will initially be safeguarded by the main investigator and will be transferred to an independent person to be kept for a longer period of time after initial processing of the data and materials. Handling of personal data will comply with the Dutch Personal Data Protection Act.

All clinical specimens that are stored will only contain the identification code, and will never contain names, initials or date of birth. Data tracking will be safeguarded by recording all stored samples in a digital freezer archive which is stored on a secured networkdrive of Erasmus MC. All samples belonging to this study will be made traceable by adding the keyword for the study (Bb2013) to each record.

The study does not involve detailed questionnaires. The only information recorded for subjects recruited to cohort A will be date of sampling, gender, age (in months), number of siblings and number of days before or after onset of rash. The only information recorded for subjects recruited to cohort B will be date of sample collection, date of onset of disease, gender, age (in months) and number of siblings.

8.2 Monitoring and Quality Assurance

A data monitoring plan has been developed to implement quality assurance. In this document, the risk classification is assessed as being negligible. Therefore, data monitoring will be performed as specified in appendix A of the monitoring plan. This requires that the study is visited at least once a year by an independent monitor, who will assess the completeness and integrity of the dossier, the samples and the data as specified in the relevant appendix.

8.3 Amendments

Amendments are changes made to the research after a favourable opinion by the accredited METC has been given. All amendments will be notified to the METC that gave a favourable opinion.

8.4 Annual progress report

The sponsor/investigator will submit a summary of the progress of the trial to the accredited METC once a year. Information will be provided on the date of inclusion of the first subject, numbers of subjects included and numbers of subjects that have completed the trial, serious adverse events/ serious adverse reactions, other problems, and amendments.

8.5 End of study report

The investigator will notify the accredited METC of the end of the study within a period of 8 weeks. The end of the study is defined as the last patient's last visit.

In case the study is ended prematurely, the investigator will notify the accredited METC within 15 days, including the reasons for the premature termination.

Within one year after the end of the study, the investigator/sponsor will submit a final study report with the results of the study, including any publications/abstracts of the study, to the accredited METC.

8.6 Public disclosure and publication policy

Once the research is completed, a summary of the results and/or any scientific publications will be filed with the assessing review committee. We will strictly adhere to the CCMO publication policy. The trial will be registered in a public trial registry before inclusion of the first patient.

9. STRUCTURED RISK ANALYSIS

Not applicable

9.1 Potential issues of concern

9.2 Synthesis

10. REFERENCES

1 Griffin DE. Measles virus. In: Knipe DM, Howley PM, eds. Fields Virology. 5 edn. Philadelphia: Lippincott Williams & Wilkins, 2007: p. 1551-85.

2 Simons E, Ferrari M, Fricks J et al. Assessment of the 2010 global measles mortality reduction goal: results from a model of surveillance data. *Lancet* 2012; **379**: 2173-8.

3 Van den Hof S, Conyn-van Spaendonck MAE, van Steenbergen JE. Measles epidemic in the Netherlands, 1999-2000. *J Infect Dis* 2002; **186**: 1483-6.

4 Lamb RA, Parks GD. *Paramyxoviridae*: the viruses and their replication. In: Knipe DM, Howley PM, eds. Fields Virology. 5 edn. Philadelphia: Lippincott Williams & Wilkins, 2007: p. 1449-96.

5 Choppin PW, Compans RW. Reproduction of paramyxoviruses. In: Fraenkel-Conrat H, Wagner RR, eds. Comprehensive virology: reproduction, large RNA viruses.New York: Plenum Press, 1975: p. 95-178.

6 Moss WJ, Griffin DE. Measles. *Lancet* 2012; **379**: 153-64.

7 Beckford AP, Kaschula RO, Stephen C. Factors associated with fatal cases of measles. A retrospective autopsy study. *S Afr Med J* 1985; **68**: 858-63.

8 Akramuzzaman SM, Cutts FT, Hossain MJ et al. Measles vaccine effectiveness and risk factors for measles in Dhaka, Bangladesh. *Bull World Health Organ* 2002; **80**: 776-82.

9 Van den Hof S, Smit C, van Steenbergen JE, De Melker HE. Hospitalizations during a measles epidemic in the Netherlands, 1999 to 2000. *Pediatr Infect Dis J* 2002; **21**: 1146-50.

10 Shanks GD, Lee SE, Howard A, Brundage JF. Extreme mortality after first introduction of measles virus to the polynesian island of Rotuma, 1911. *Am J Epidemiol* 2011; **173**: 1211-22.

11 Duke T, Mgone CS. Measles: not just another viral exanthem. *Lancet* 2003; **361**: 763-73.

12 Moss WJ, Griffin DE. Global measles elimination. *Nat Rev Microbiol* 2006; **4**: 900-8.

13 Moss WJ. Measles still has a devastating impact in unvaccinated populations. *PLoS Med* 2007; **4**: e24.

14 Van Binnendijk RS, van der Heijden RWJ, Van Amerongen G, UytdeHaag FGCM, Osterhaus ADME. Viral replication and development of specific immunity in macaques after infection with different measles virus strains. *J Infect Dis* 1994; **170**: 443-8.

15 Kobune F, Takahashi H, Terao K et al. Nonhuman primate models of measles. *Lab Anim Sci* 1996; **46**: 315-20.

16 McChesney MB, Miller CJ, Rota PA et al. Experimental measles I. Pathogenesis in the normal and the immunized host. *Virology* 1997; **233**: 74-84.

17 Auwaerter PG, Rota PA, Elkins WR et al. Measles virus infection in rhesus macaques: altered immune responses and comparison of the virulence of six different virus strains. *J Infect Dis* 1999; **180**: 950-8.

18 De Swart RL. Measles studies in the macaque model. *Curr Top Microbiol Immunol* 2009; **330**: 55-72.

19 Remfry J. A measles epizootic with 5 deaths in newly-imported rhesus monkeys (Macaca Mulatta). *Lab Anim* 1976; **10**: 49-57.

20 Ludlow M, Duprex WP, Cosby SL, Allen IV, McQuaid S. Advantages of using recombinant measles viruses expressing a fluorescent reporter gene with vibratome slice technology in experimental measles neuropathogenesis. *Neuropathol Appl Neurobiol* 2008; **34**: 424-34.

21 De Swart RL, Ludlow M, De Witte L et al. Predominant infection of CD150+ lymphocytes and dendritic cells during measles virus infection of macaques. *PLoS Pathog* 2007; **3**: e178.

22 Lemon K, De Vries RD, Mesman AW et al. Early target cells of measles virus after aerosol infection of non-human primates. *PLoS Pathog* 2011; **7**: e1001263.

23 Ludlow M, Lemon K, De Vries RD et al. Measles virus infection of epithelial cells in the macaque upper respiratory tract is mediated by sub-epithelial immune cells. *J Virol* 2013; **87**: 4033-42.

24 Ludlow M, De Vries RD, Lemon K et al. Infection of lymphoid tissues in the macaque upper respiratory tract contributes to the emergence of transmissible measles virus. *J Gen Virol* 2013; **in press**.

25 Griffin DE, Ward BJ, Jauregui E, Johnson RT, Vaisberg A. Immune activation in measles. *N Engl J Med* 1989; **320**: 1667-72.

26 Akramuzzaman SM, Cutts FT, Wheeler JG, Hossain MJ. Increased childhood morbidity after measles is short-term in urban Bangladesh. *Am J Epidemiol* 2000; **151**: 723-35.

27 Von Pirquet CE. Das Verhalten der kutanen Tuberkulin-reaktion während der Masern. *Dtsch Med Wochenschr* 1908; **34**: 1297-300.

28 Tamashiro VG, Perez HH, Griffin DE. Prospective study of the magnitude and duration of changes in tuberculin reactivity during uncomplicated and complicated measles. *Pediatr Infect Dis J* 1987; **6**: 451-4.

29 Lisse I, Samb B, Whittle H et al. Acute and long-term changes in T-lymphocyte subsets in response to clinical and subclinical measles. A community study from rural Senegal. *Scand J Infect Dis* 1998; **30**: 17-21.

30 Ryon JJ, Moss WJ, Monze M, Griffin DE. Functional and phenotypic changes in circulating lymphocytes from hospitalized Zambian children with measles. *Clin Diagn Lab Immunol* 2002; **9**: 994-1003.

31 Premenko-Lanier M, Rota PA, Rhodes GH, Bellini WJ, McChesney MB. Protection against challenge with measles virus (MV) in infant macaques by an MV DNA vaccine administered in the presence of neutralizing antibody. *J Infect Dis* 2004; **189**: 2064-71.

32 Bankamp B, Hodge G, McChesney MB, Bellini WJ, Rota PA. Genetic changes that affect the virulence of measles virus in a rhesus macaque model. *Virology* 2008; **373**: 39-50.

33 Hirsch RL, Griffin DE, Johnson RT et al. Cellular immune responses during complicated and uncomplicated measles virus infections of man. *Clin Immunol Immunopathol* 1984; **31**: 1-12.

34 Ward BJ, Johnson RT, Vaisberg A, Jauregui E, Griffin DE. Cytokine production in vitro and the lymphoproliferative defect of natural measles virus infection. *Clin Immunol Immunopathol* 1991; **61**: 236-48.

35 Avota E, Avots A, Niewiesk S et al. Disruption of Akt kinase activation is important for immunosuppression induced by measles virus. *Nat Med* 2001; **7**: 725-31.

36 Griffin DE, Ward BJ. Differential CD4 T cell activation in measles. *J Infect Dis* 1993; **168**: 275-81.

37 Ward BJ, Griffin DE. Changes in cytokine production after measles virus vaccination: predominant production of IL-4 suggests induction of a Th2 response. *Clin Immunol Immunopathol* 1993; **67**: 171-7.

38 Fugier-Vivier I, Servet-Delprat C, Rivailler P et al. Measles virus suppresses cell-mediated immunity by interfering with the survival and functions of dendritic and T cells. *J Exp Med* 1997; **186**: 813-23.

39 Grosjean I, Caux C, Bella C et al. Measles virus infects human dendritic cells and blocks their allostimulatory properties for CD4+ T cells. *J Exp Med* 1997; **186**: 801-12.

40 Schnorr J-J, Xanthakos S, Keikavoussi P et al. Induction of maturation of human blood dendritic cell precursors by measles virus is associated with immmunosuppression. *Proc Natl Acad Sci USA* 1997; **94**: 5326-31.

41 Condack C, Grivel J-C, Devaux P, Margolis L, Cattaneo R. Measles virus vaccine attenuation: suboptimal infection of lymphatic tissue and tropism alteration. *J Infect Dis* 2007; **196**: 541-9.

42 De Vries RD, McQuaid S, Van Amerongen G et al. Measles immune suppression: lessons from the macaque model. *PLoS Pathog* 2012; **8**: e1002885.

43 Sallusto F, Lenig D, Forster R, Lipp M, Lanzavecchia A. Two subsets of memory T lymphocytes with distinct homing potentials and effector functions. *Nature* 1999; **401**: 708-12.

44 Ruijs WLM. Acceptance of vaccination among orthodox protestants in the Netherlands. Nijmegen: Radboud University, 2012.

45 Van den Hof S, Meffre CM, Conyn-Van Spaendonck MA et al. Measles outbreak in a community with very low vaccine coverage, the Netherlands. *Emerg Infect Dis* 2001; **7**: 593-7.

46 Hahné S, te Wierik MJM, Mollema L et al. Measles outbreak, the Netherlands, 2008. *Emerg Infect Dis* 2010; **16**: 567-9.

47 Van Binnendijk RS, Van den Hof S, Van den Kerkhof H et al. Evaluation of serological and virological tests in the diagnosis of clinical and subclinical measles virus infections during an outbreak of measles in The Netherlands. *J Infect Dis* 2003; **188**: 898-903.
